# WRN modulates translation by influencing nuclear mRNA export in HeLa cancer cells

**DOI:** 10.1186/s12860-020-00315-9

**Published:** 2020-10-14

**Authors:** Juan Manuel Iglesias-Pedraz, Diego Matia Fossatti-Jara, Valeria Valle-Riestra-Felice, Sergio Rafael Cruz-Visalaya, Jose Antonio Ayala Felix, Lucio Comai

**Affiliations:** 1 Departamento de Investigación, Desarrollo e Innovación, Laboratorio de Genética Molecular y Bioquímica, Universidad Científica del Sur, Villa El Salvador, 15842 Lima, Peru; 2 Present address: National Centre for Biomolecular Research, Masaryk University, 62500 Brno, Czech Republic; 3 Department of Molecular Microbiology and Immunology, Biochemistry and Molecular Medicine, Keck School of Medicine, Longevity Institute, Davis School of Gerontology, University of Southern California, Los Angeles, CA 90033 USA

**Keywords:** Werner syndrome protein, mRNA export, NXF1 export receptor, Translation, Cancer, Senescence

## Abstract

**Background:**

The Werner syndrome protein (WRN) belongs to the RecQ family of helicases and its loss of function results in the premature aging disease Werner syndrome (WS). We previously demonstrated that an early cellular change induced by WRN depletion is a posttranscriptional decrease in the levels of enzymes involved in metabolic pathways that control macromolecular synthesis and protect from oxidative stress. This metabolic shift is tolerated by normal cells but causes mitochondria dysfunction and acute oxidative stress in rapidly growing cancer cells, thereby suppressing their proliferation.

**Results:**

To identify the mechanism underlying this metabolic shift, we examined global protein synthesis and mRNA nucleocytoplasmic distribution after WRN knockdown. We determined that WRN depletion in HeLa cells attenuates global protein synthesis without affecting the level of key components of the mRNA export machinery. We further observed that WRN depletion affects the nuclear export of mRNAs and demonstrated that WRN interacts with mRNA and the Nuclear RNA Export Factor 1 (NXF1).

**Conclusions:**

Our findings suggest that WRN influences the export of mRNAs from the nucleus through its interaction with the NXF1 export receptor thereby affecting cellular proteostasis. In summary, we identified a new partner and a novel function of WRN, which is especially important for the proliferation of cancer cells.

## Background

Werner Syndrome (WS) is an autosomal recessive disorder of premature aging [1, 2]. WS is caused by mutations in a single gene located on chromosome 8 [3], which encodes a nuclear protein termed Werner syndrome protein (WRN) [4]. WRN has helicase and exonuclease activity [5–7], and is one of four human RecQ helicases that function as genome caretakers [8]. WS patients display a striking predisposition to the early development of a range of diseases, which are typically observed in older individuals during normal aging. These include atherosclerosis, osteoporosis, type II diabetes mellitus, and predisposition to several types of cancer, with a higher prevalence of mesenchymal malignancies (sarcomas) and relatively low incidence of carcinomas [2, 9–14]. WS-derived fibroblasts compared with fibroblasts derived from normal patients show a dramatic decrease in the proliferation rate together with an accumulation of oxidative DNA damage, leading to premature replicative senescence [15–18]. Since WRN is undetectable in WS cells, the mutant mRNA is thought to be degraded by nonsense-mediated mRNA decay (NMD) [19, 20]. Thus, WS is considered a loss-of-function genetic disease.

Although genome instability and premature senescence of primary fibroblasts derived from WS patients are suppressed by expression of telomerase, depletion of WRN inhibits the proliferation of many of telomerase-positive cancer cell lines [21, 22] indicating that WRN has an intrinsic growth promoting activity that is not complemented by telomerase. Moreover, several studies have shown that WRN is markedly upregulated in many tumor cells and cell lines [21–23]. Consistent with the idea that WRN is required for cancer cell proliferation, WRN knockdown suppresses anchorage-dependent growth of cancer cells in vitro and MYC-induced oncogenesis in animal models [21, 24]. To gain an understanding of the role of WRN in cancer cell proliferation, we used a conditional shRNAs system to precisely define the early cellular changes induced by WRN depletion. Our data demonstrated that WRN depletion results in a significant decrease in the level of enzymes involved in metabolic pathways, including glucose 6-phosphate dehydrogenase (G6PD) that control macromolecular synthesis and protect cells from oxidative stress soon after WRN depletion [25]. This metabolic shift is tolerated by normal cells but causes acute oxidative stress overload in rapidly growing cancer cells, which suppresses their proliferation and further analysis suggested that the metabolic changes induced by WRN depletion are caused by attenuated translation [25].

The increased proliferation of tumor cells requires increased rates of protein synthesis, which requires alterations in pathways that promote translation [26, 27]. Newly synthesized precursors mRNAs (heterogenous RNA, hnRNAs), undergo a series of processing steps [28, 29] before they assemble into a mature messenger ribonucleoprotein particle (mRNP) [28, 30, 31]. mRNPs, through association with export receptors including nuclear export factor 1 (NXF1 or tip-associated protein (TAP)) and chromosome maintenance protein 1 (CRM1, also known as exportin 1; XPO1, [32]) are transported into the cytoplasm through the nuclear pore complex for translation [33, 34]. Interestingly, WRN has been shown to interact with the nuclear pore protein Nup107 as well as other nucleoporins located at the inner ring complex or center of the channel [35]. Although the functional role of these interactions is poorly understood, they suggest a potential function of WRN at the nuclear periphery.

In this study we investigated the mechanism of translational attenuation by WRN depletion using HeLa cells as a cancer cell model and demonstrate that WRN influences the nucleocytoplasmic distribution of mRNAs. Remarkably, we found that WRN directly interacts with the export receptor NXF1 and co-precipitates with mRNAs. These data indicate that WRN contributes to the nuclear export of mRNA and reduced export of mRNAs upon WRN depletion is likely responsible for attenuated synthesis of proteins including enzymes involved in redox homeostasis that are critical for the proliferation of many cancer cells.

## Results

### WRN depletion results in decreased levels of de-novo protein synthesis

**Fig. 1:**
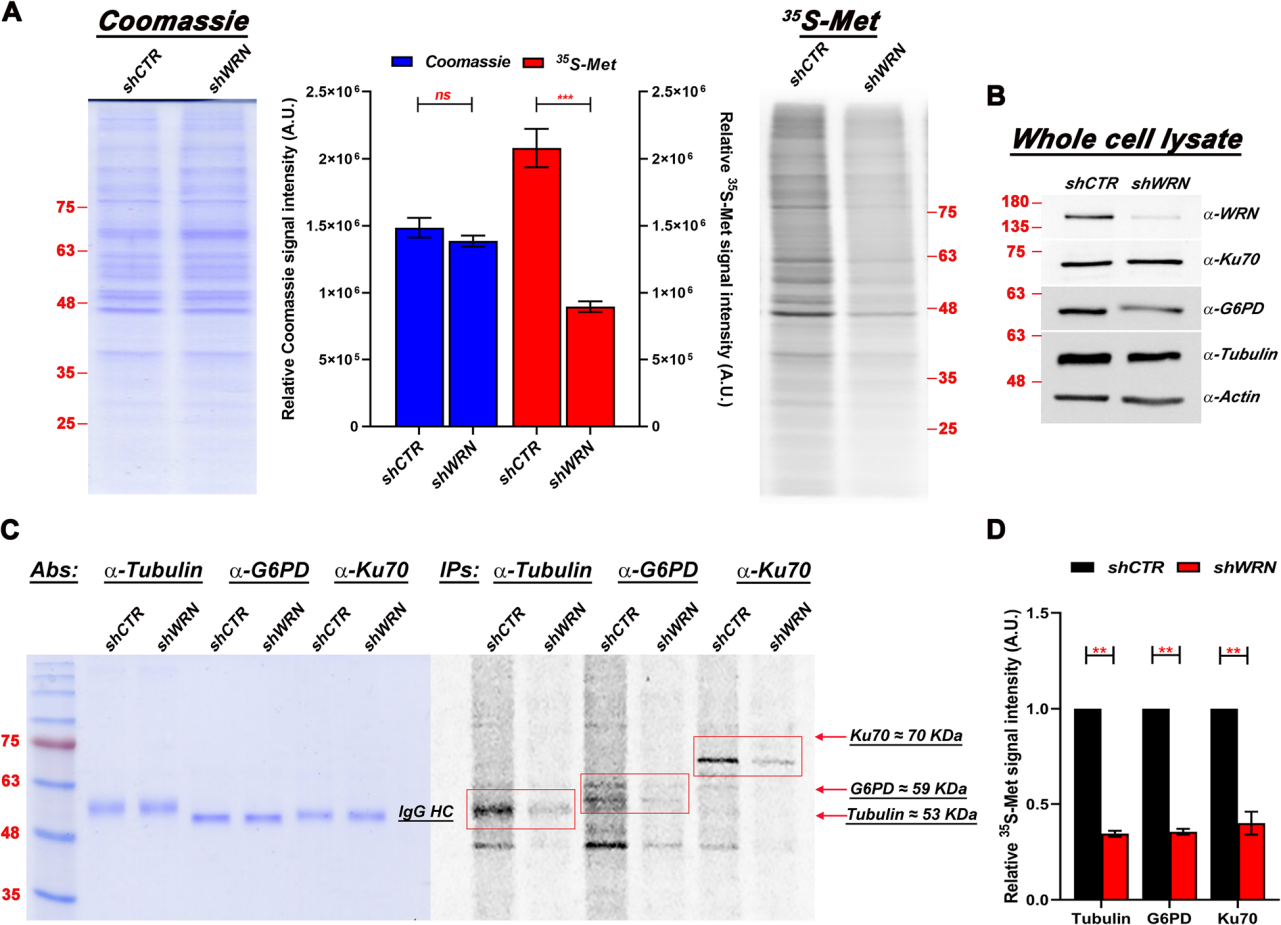
WRN depletion affects *de-novo* synthesis of proteins. **a** Pulse-labeling of shCTR and WRN-depleted HeLa cells 3 days after shRNA induction. The same number of cells were used to prepare the lysates and radiolabeled methionine incorporation (right panel) was visualized as described in the Methods section and Supplemental Information. Coomassie stained gels of total proteins (left panel) was used to confirm equal loading. Data plotted represent relative intensities ± SEM and Two-way ANOVA followed by Sidak’s multiple comparisons test from GraphPad Prism (ns, no significant difference; *** *p* < 0.001) of three biological replicates. **b** Representative Western blot showing WRN downregulation 72 h after shRNA induction. Molecular size markers (in KiloDaltons) are shown. **c** as in (**a**) using the indicated antibodies. Equal amounts of antibody used in each IP reaction was confirmed by Coomassie staining (left panel). The amount of each immunoprecipitated radiolabeled protein (red squares, right panel) in shCTR and shWRN extracts was normalized against immunoglobulin heavy chain (IgG HC) from the Coomassie-stained gel. The experiment shown is a representative of three biological replicates. **d** Plot of the normalized band intensities of ^35^S generated represent relative intensities ± SEM and Two-way ANOVA followed by Sidak’s multiple comparisons test from GraphPad Prism (** *p* < 0.01) of three biological replicates

Our previous work suggested that the metabolic changes induced by WRN depletion in cancer cells are caused by a posttranscriptional mechanism of deregulation [25], and we confirmed by RT-qPCR that mRNA levels of the metabolic enzymes G6PD and IDH1 are not downregulated after shWRN induction in HeLa cancer cells (Supplementary Fig. 1A). To gain an understanding of the processes that are responsible for the metabolic changes observed in cancer cells upon WRN depletion [25], we measured *de-novo* protein synthesis by metabolic pulse labeling experiments with [^35^S]-methionine/cysteine (^35^S-met/cys) in HeLa cells after the induction of shWRN or control shRNAs. We analyzed the cells after three days of shRNA induction since we have previously shown that this is the earliest time point with more than 80% depletion of WRN. The extracted proteins were resolved by polyacrylamide-gel electrophoresis (SDS-PAGE) and visualized by Coomassie stain. The dried gels were then exposed to a phosphorimaging screen to visualize the *de-novo* synthesized radiolabeled proteins. The results of these experiments show a significant decline in radiolabeled proteins in WRN-depleted cells (~ 43%; *p* < 0.001) as compared to the control cells (Fig. 1a and b), indicating that loss of WRN negatively affects *de-novo* protein synthesis.

To specifically determine whether the decrease in the levels of the metabolic enzyme G6PD after WRN depletion is the result of reduced rates of protein synthesis, the extracts prepared from pulse-labeled WRN-depleted and control cells were subjected to immunoprecipitation (IP) with G6PD antibody. The immunoprecipitated products were resolved on an SDS-PAGE and Coomassie stain was used to visualize the immunoglobulin heavy chain, as a means to assure that equal amounts of antibody were added to each reaction. The level of radiolabeled G6PD was measured by exposing the gel to a phosphorimaging screen. The radiolabeled met/cys incorporated in *de-novo* synthesized G6PD in WRN-depleted HeLa cells was plotted relative to control cells and normalized to Coomassie signal intensity of the antibody heavy chain. The results of these experiments show a significant decrease in the levels of radiolabeled G6PD in WRN depleted cells as compared to the control cells (Fig. 1c), indicating that reduced translation of G6PD mRNA is likely contributing to the reduced levels of G6PD protein observed in these cells [25].

Since the results shown in Fig. 1a suggest that WRN depletion has a more general effect on *de-novo* protein synthesis, we then performed immunoprecipitation reactions using antibodies against two arbitrarily selected proteins, tubulin and Ku70. These experiments show reduced levels of *de-novo* synthesized tubulin and Ku70 in WRN depleted cells (Fig. 1c and d), a result that is consistent with WRN depletion having a rather general effect on protein biosynthesis. To rule out that reduced levels of *de-novo* protein synthesis is the result of a stress response, we analyzed stress granules (SGs) formation in WRN-depleted and control cells by immunofluorescence microscopy using an antibody against the RNA-binding protein TIAR. In response to environmental stress, including oxidative conditions, TIAR accumulates into the cytoplasm and aggregates at SGs [36–38]. The formation of SGs is closely linked to the inhibition of translation initiation [39]. The results show that TIAR aggregation is not detected after WRN depletion (Supplementary Fig. 1B and C). Importantly, the formation of SGs is not inherently inhibited by WRN depletion, since discrete cytoplasmic foci are formed in WRN depleted treated with arsenite, a known inducer of SGs [40] (Supplementary Fig. 1C).

We have previously shown that DNA damage and the ensuing response occur at later time points (approximately 5 days) after WRN depletion than the downregulation of metabolic enzymes [25, 41] and confirmed that three days after WRN knockdown there is no significant increase in the levels of the phosphorylated form of γ-H2AX, an established marker of DNA breaks (Supplementary Fig. 1D and E) [17, 42], in HeLa cells. Collectively, these results rule out that activation of a stress response pathways is the cause of attenuated translation after WRN depletion.

### WRN depletion does not impact the levels of ribosomal proteins (RPs) nor ribosomal RNA (rRNA)

**Fig. 2:**
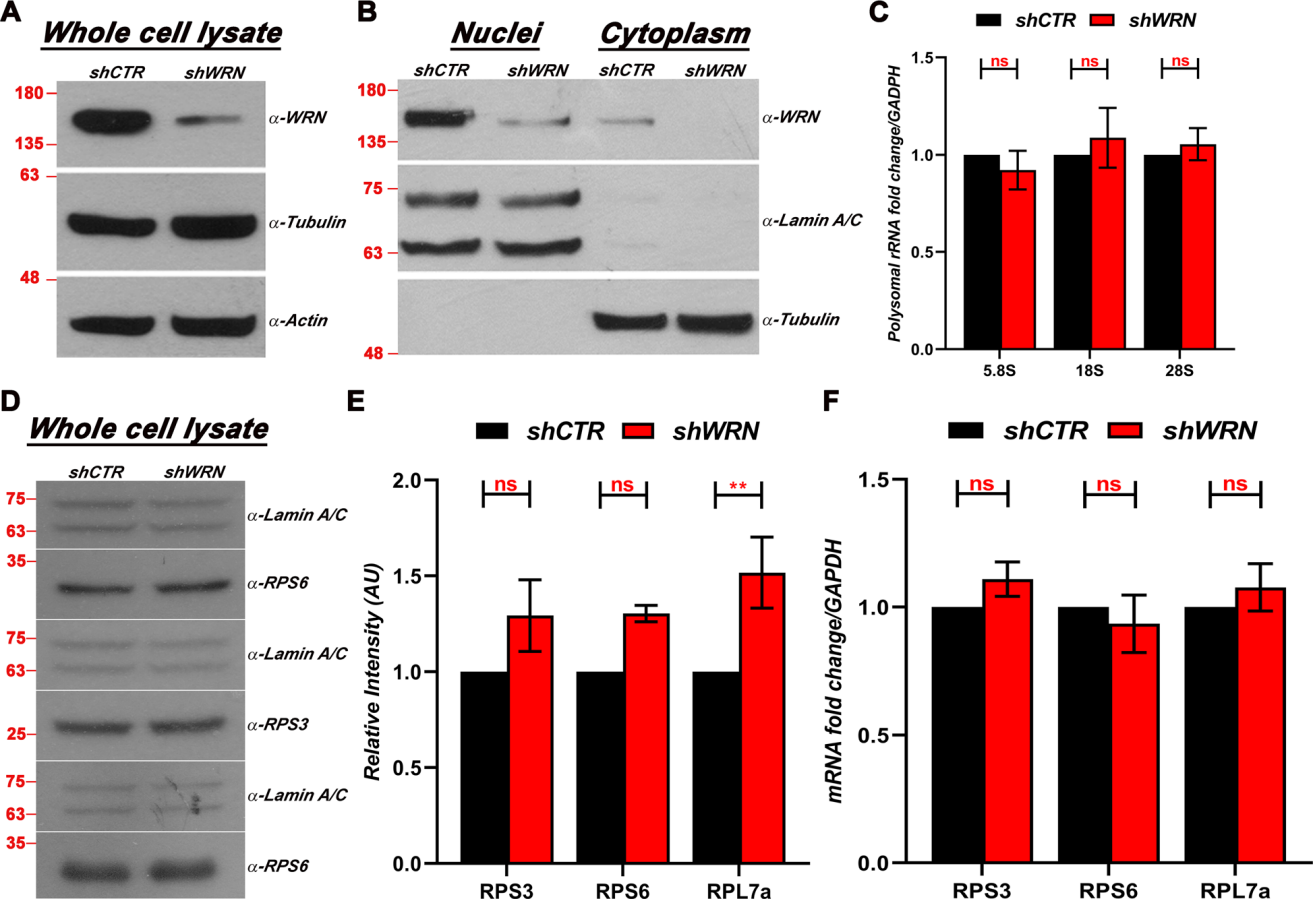
WRN depletion does not affect the levels of ribosomal proteins or ribosomal rRNAs. **a** A representative Western blot of WRN depletion after 3 days of dox treatment using actin as loading control. **b** Representative Western blot of nuclear-cytoplasmic fractionation of HeLa cells. The absence of cross contamination between nuclear and cytoplasmic fractions was confirmed using Lamin A/C (nuclear marker) and Tubulin (cytoplasmic marker). WRN depletion was confirmed in the same blot. Molecular size markers (in KiloDaltons) are shown. **c** Total RNA was purified from the Polysome fractions as described in the Methods and the Supplemental Information sections, and used for quantitative RT-qPCR analysis with sets of primers against 5.8S, 18S and 28S rRNAs. GAPDH was used as the internal control. The significance of the fold changes in three biological replicates was calculated using two-way ANOVA followed by Sidak’s multiple comparisons test from GraphPad Prism. The error bars represent the mean ± SEM (*n* = 3). ns, no significant difference. **d** A representative Western blot analysis of whole cell lysates derived from shCTR control and WRN-depleted cells 72 h after dox induction using antibodies against the indicated ribosomal proteins. Molecular size markers (in KiloDaltons) are shown for both experiments. **e** Band intensity determination in three biological replicates was generated using ImageJ (NIH) and plotted using GraphPad Prism. **f** Quantitative RT-qPCR analysis of ribosomal proteins genes using GAPDH as the internal control was performed as previously described, and the results of three biological replicates were plotted using GraphPad Prism. **e** and **f**, Two-way ANOVA followed by Sidak’s multiple comparisons test from GraphPad Prism, was used to calculate the significance in both approaches. The error bars represent the mean ± SEM (*n* = 3). ** *p* value < 0.01; ns, no significant differences

Ribosomes are complex structures composed of two main components, the 40S, and 60S subunits. Both subunits are organized by ribosomal RNA (rRNA), proteins, and accessories factors [43]. The large subunit (60S) consists of the 28S, 5S and 5.8S rRNAs and 47 proteins, while the small subunit (40S) has a single 18S rRNA and 33 proteins [44–46]. It has been reported that WS fibroblasts show decreased levels of rRNA transcription compared with wild-type cells, which was reversed by ectopic expression of wild-type WRN [47]. Since this defect could directly affect protein synthesis, we wanted to determine if attenuated translation is the result of changes in the abundance of rRNAs in mature ribosomes after WRN depletion in HeLa cells. Polysome extracts from WRN depleted and control cells were prepared by sucrose gradient differential centrifugation of purified cytoplasmic fractions (Fig. 2a and b, and Supplementary Fig. 2A and B). After RNAs purification, quantitative RT-qPCR analysis was performed using primers sets for the 28S, 18S, and 5.8S rRNAs. The results of this experiment do not show any significant difference in rRNA abundance between WRN depleted and control HeLa cells (Fig. 2c). This result suggests that attenuated protein synthesis during the early phase of WRN depletion is not the result of altered rRNA levels in mature ribosomes of HeLa cells.

Next, we analyzed the levels of a subset of ribosomal proteins (RPs) and their cognate mRNAs in WRN depleted and control HeLa whole cell lysates. We performed semi-quantitative analysis of a set of ribosomal proteins using Lamin A/C as a normalizing protein [48] and did not observed any detectable decrease in the expression levels of the analyzed RPs (Fig. 2d). Rather, WRN-depleted cells show a significant increase in the signal intensity of these RPs (Fig. 2e), possibly indicating a compensation mechanism in response to decreased translation. Analysis by qPCR did not show any difference in the steady-state levels of RPs mRNAs between WRN depleted and control samples (Fig. 2f), suggesting that the observed increase in protein levels might be the result of increased proteins stability. Taken together, these data indicate that the decrease in *de-novo* protein synthesis observed in WRN depleted cells is not due to lower abundance of ribosomal components of the translational machinery.

### WRN depletion affects the nuclear/cytoplasmic distribution of mRNAs

**Fig. 3:**
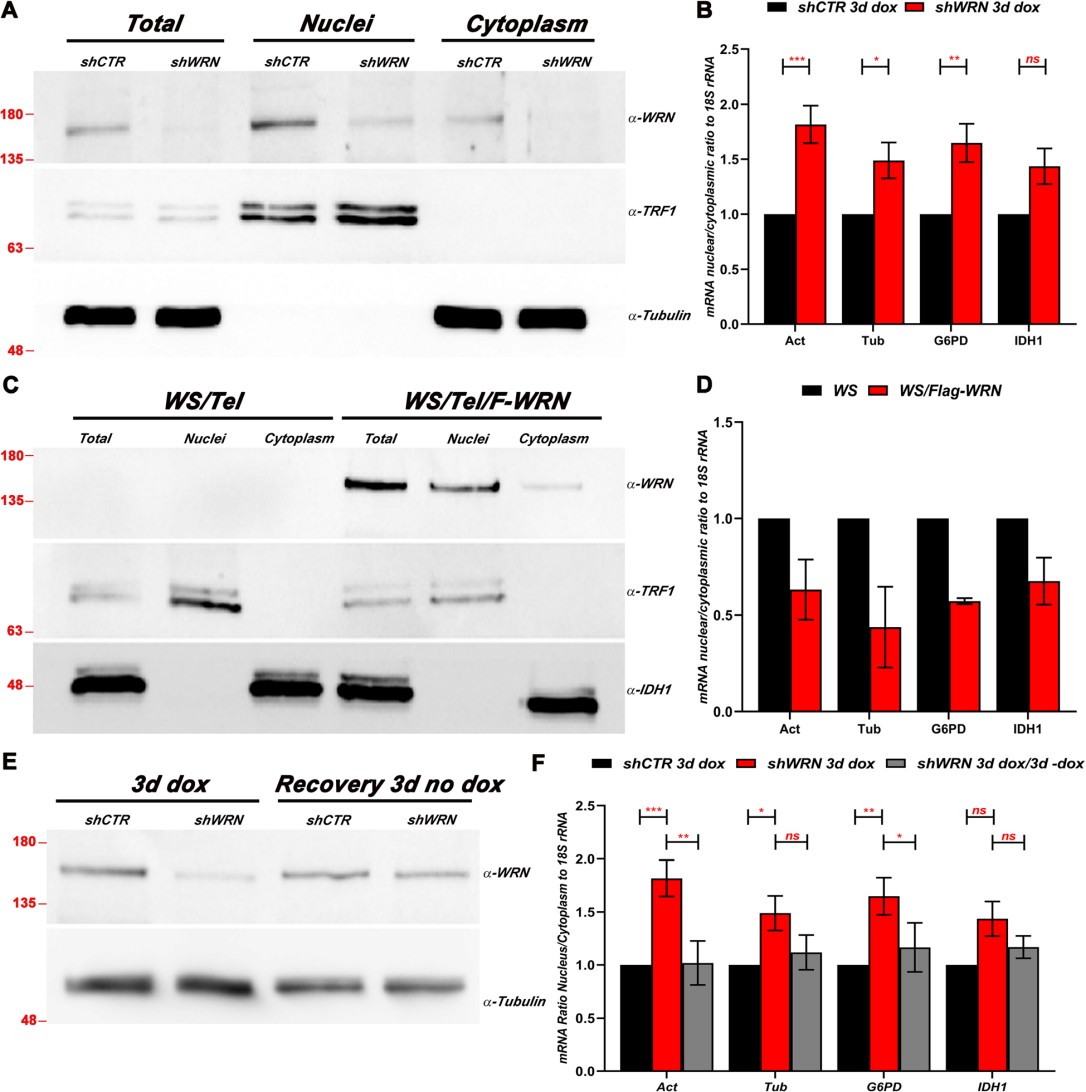
RT-qPCR reveals spatial distributions alterations of mRNAs in HeLa cells and in WS fibroblast. **a** HeLa cells were lysed as described in the Methods and the Supplemental Information sections to obtain nuclear and cytoplasmic fractions. Anti-TRF2 antibody was used to assure that cytoplasmic extracts were not contaminated with nuclear proteins. Molecular size markers (in KiloDaltons) are shown. **b** Total RNA was extracted from each fraction and analyzed by RT-qPCR using the indicated set of primers (Supplementary Table 2). The results of nuclear/cytoplasmic ratio were plotted using GraphPad Prism. **c** WS patient-derived fibroblasts and the derivative cell line with ectopic expression of flag-WRN (WS/F-WRN) were lysed as in (**a**) to obtain nuclear and cytoplasmic fractions. Molecular size markers (in KiloDaltons) are shown. **d** Total RNA was extracted from each fraction and analyzed by RT-qPCR using primers sets as in (**b**) and the results were plotted using GraphPad Prism. **e** Cells were first incubated in media containing dox for three days. At day three, the cells were washed gently and incubated for 3 additional days in media without dox. Next, the cells were lysed, and extract were processed as describe in (**a**). Western blot analysis was used to assess WRN expression. Molecular size markers (in KiloDaltons) are shown. **f** The nuclear and cytoplasmic fractions were used to isolated total RNA, which was analyzed by RT-qPCR with primers for the indicated transcripts. In (**b**) and (**f**) a Two-way ANOVA followed by Sidak’s multiple comparisons test was used to calculate only the significance between shCTR and shWRN HeLa cells after dox induction. The error bars represent the mean ± SEM (n = 3). * *p* value < 0.05; ** *p* value < 0.001; *** *p* value ≤0.0002; ns, no significant differences. In (**d**) the experiment was performed in two biological replicates, thus the means and ± SEM without statistical analysis are shown

The aforementioned experiments led us to then test whether the dysfunction in protein synthesis may be caused by alteration in mRNA nucleocytoplasmic transport. mRNA export is a critical step in promoting gene expression and upregulation of the mRNA export factors has been observed in many types of cancer [22, 23, 49]. We isolated nuclear and cytoplasmic fractions from equal number of WRN depleted and control HeLa cells and measured the mRNA present in each fraction by RT-qPCR. Prior to running the qPCR reactions, each fraction was analyzed by SDS-PAGE and immunoblotted using nuclear and cytoplasmic protein markers to ensure no cross-contamination between these two compartments (Fig. 3a). We performed qPCR reactions using primer sets for actin, tubulin, G6PD and IDH1 and observed altered nuclear to cytoplasmic mRNA ratio for all these transcripts in WRN depleted cells when compared to the control (Fig. 3b). To assess the generality of our observation and rule out potential effects caused by the shRNA silencing system used in this analysis, we compared nuclear/cytoplasmic ratios of the same set of mRNAs between parental WS fibroblasts and WS fibroblasts reconstituted with Flag-tagged WRN (F-WRN). The results of this experiment show that the cells reconstituted with Flag-WRN display a lower nuclear/cytoplasmic mRNA ratio compared to the parental cells (Fig. 3c and d), indicating that this process is also affected in normal cells. However, because of the lower proliferation rates of fibroblasts, the effects on these cells are likely to manifest after many more cell divisions. Consistent with these data, the alterations in the nuclear/cytoplasmic mRNA ratio associated with WRN depletion in HeLa cells were in part reversed after removal of doxycycline, which allows re-expression of WRN (Fig. 3e and f).

**Fig. 4:**
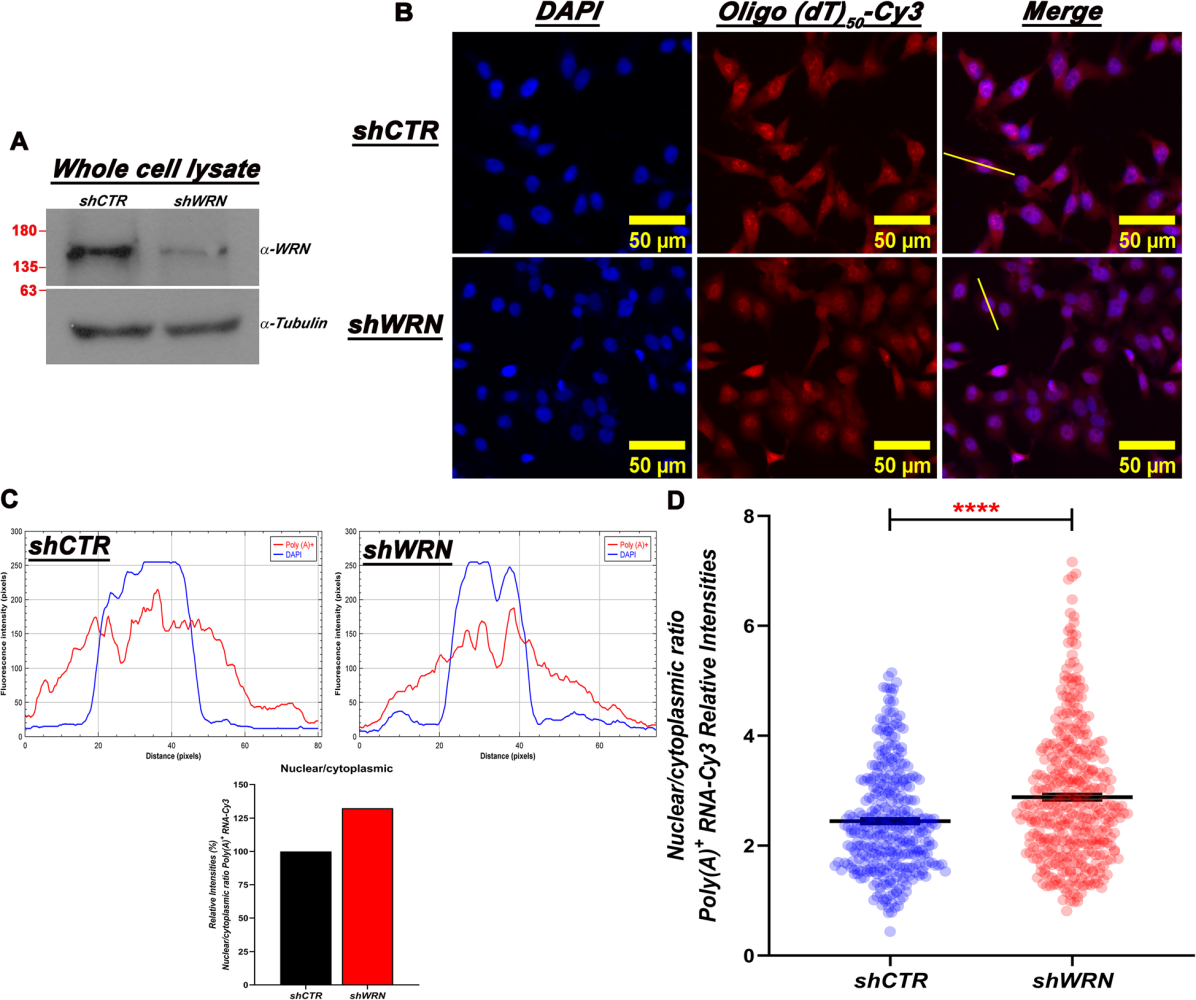
FISH analysis shows a significative change in mRNA distribution after WRN depletion in HeLa cells. **a** Representative Western blot showing WRN levels in shCTR and shWRN HeLa cells after 72 h of dox treatment. Molecular size markers (in KiloDaltons) are shown. **b** shCTR and shWRN HeLa cells were fixed and hybridized with Oligo (dT)_50_-Cy3 as described in Supplemental Information. Representative images showing the mRNA signal in each cell line (Oligo (dT)_50_-Cy3) and the yellow line drawn across cells used to measure the fluorescence intensity in pixels (Scale bar = 50 μm). **c** The nuclear periphery (as defined by DAPI staining) was used to separate the intensities of the cytoplasmic and nuclear Poly (A)^+^ signal and the obtained data were plotted using ImageJ. The sum of the resulting intensities from the nuclei and cytoplasm quantitation were used to calculate the nuclear/cytoplasm mRNA ratio in shCTR and shWRN cell and plotted using GraphPad Prism (bottom graph). **d** For each cell analyzed, the average of the fluorescence intensity (pixels) in the nuclei was divided by the average of the fluorescence intensity (pixels) of the cytoplasm and the resulting data was used to generate the graph. Two-tailed unpaired *t* test from GraphPad Prism was used to calculate the significance between WRN-depleted and control cells. The error bars represent the mean ± SEM of three independent experiments (shCTR *n* = 342 cells, shWRN *n* = 389). **** *p* value < 0.0001

To confirm the alteration in the mRNA nuclear/cytoplasmic distribution, control and WRN-depleted HeLa cells were analyzed by mRNA Fluorescent In Situ Hybridization (FISH) using a conjugated-Cy3 Oligo (dT) probe. Three days after doxycycline treatment the cells were fixed, incubated with the Oligo (dT) probe and analyzed by confocal microscopy. A fraction of the cells was used to confirm WRN depletion by immunoblot (Fig. 4a). The signal intensity of the Oligo (dT) Cy3-conjugated probe was measured in the cell-fixed images by tracing a pixel-fixed line across the entire cell and the nuclear/cytoplasmic boundary was determined at the intersection of the DAPI and Cy3 channel (Fig. 4b and c). After measuring the individual intensities in the Cy3 channel, the nuclear/cytoplasmic ratio was calculated for the WRN-depleted and control cells. This analysis shows a significant increase in the nuclear/cytoplasmic ratio of the poly (A)^+^ signal in WRN depleted cells when compared to the controls (Fig. 4d), reinforcing the conclusion that WRN depletion affects the subcellular distribution of mRNAs in HeLa cells.

### The protein levels of components of the mRNA export pathway are not affected by WRN depletion in HeLa cells

**Fig. 5:**
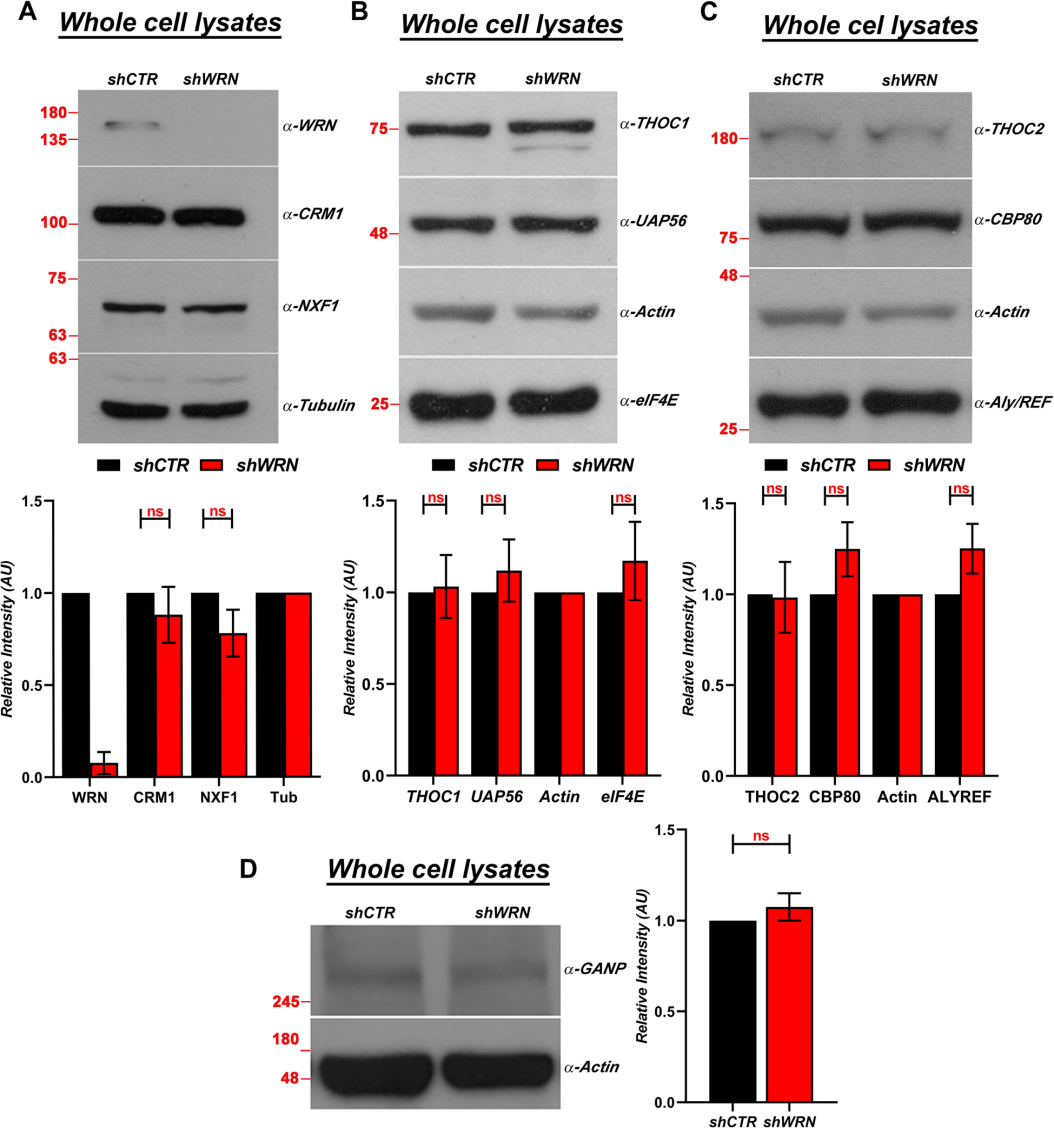
WRN depletion in HeLa cells does not results in altered levels of the major mRNA export receptors. **a** Extracts prepared from shCTR and shWRN HeLa cells 72 h after dox induction were analyzed by Western blot using the indicated antibodies. Molecular size markers (in KiloDaltons) are shown. (Lower panel) Bands intensities in four biological replicates were quantitated using ImageJ (NIH) and plotted into the graph. Two-way ANOVA followed by Sidak’s multiple comparisons test was used to calculate the significance of the means and normalized against tubulin using GraphPad Prism. The error bars represent the mean ± SEM (*n* = 4).; ns, no significant difference. **b** Western blot showing the TREX-1 subunit THOC1, UAP56 and eIF4E in WRN-depleted and control HeLa cells. (Lower panel) semiquantitative analysis of protein levels as described in (**a**). **c** Western blot showing the TREX-1 component THOC2, ALYREF and CBP80 in WRN-depleted and control HeLa cells. (Lower panel) semiquantitative analysis of protein levels as described in (**a**). **d** Western blot analysis of the mRNA export receptor GANP in WRN-depleted and control HeLa cells. Right panel shows semiquantitative analysis of protein levels as described in (**a**). In the plots of Figs. (**b**), (**c**) and (**d**) Two-way ANOVA followed by Sidak’s multiple comparisons test were used to calculate the significance of the means and normalized against tubulin using GraphPad Prism. The error bars represent the mean ± SEM (*n* = 3). ns, no significant difference. Molecular size markers (in KiloDaltons) are shown

Our data suggested that WRN depletion affects the mRNA export pathway. Therefore, we determined whether changes in the levels of the export receptors could be responsible for these alterations. We first measured the levels of the two major nuclear export factors NXF1 and CRM1 using semi-quantitative Western blot analysis. The results of this experiment show no significant difference in the levels of these two proteins between control and WRN-knockdown HeLa cells, suggesting that alterations in mRNA export after WRN depletion are unlikely caused by lower levels of either one of these proteins (Fig. 5a). Next, we examined the Transcription and Export 1 (TREX-1) factor. TREX-1 is a conserved multiprotein complex that plays a critical role in mRNPs biogenesis and maturation in eukaryotes [50–55]. This large complex links processing and export of mRNAs and provides a surveillance platform for maintaining the high-fidelity of the gene expression [56]. Several studies have shown that inhibition of TREX-1 components results in the accumulation of mRNPs in the nucleus [51, 57]. To determine whether alteration in the mRNA spatial distribution resulting from WRN depletion may be caused by reduced levels of the TREX-1 complex, we performed immunoblotting using antibodies against factors within the THO complex subcomplexes THOC1 and THOC2, including the adaptor protein ALYREF. In this analysis we also examined the levels of other factors linked to mRNA metabolism, including UAP56 and CBP80, two proteins implicated in the splicing and capping of the mRNAs, respectively, GANP (Germinal center–Associated Nuclear Protein), a protein that is actively involved in recruitment and transport of mRNPs and whose depletion causes nuclear accumulation of Poly (A)^+^ RNA [58], and eIF4E, a eukaryotic translation initiation factor that has been shown to facilitate nuclear export of specific transcripts [59, 60]. The results of this experiment show that depletion of WRN does not result in a decrease in the levels of any of these proteins as compared to the control cells (Fig. 5b-d). Interestingly, as we observed for the RPs, some of these adaptor proteins are slightly upregulated, possibly as a compensatory response to the reduced nuclear export of mRNA. Taken together, these results rule out deficiencies in components of export machinery or mRNA processing as an early response to WRN depletion and suggest that a different mechanism is likely responsible for reduced levels of *de-novo* protein synthesis in WRN depleted HeLa cells.

### WRN associates with mRNA through direct interaction with the mRNA export receptor NXF1

**Fig. 6:**
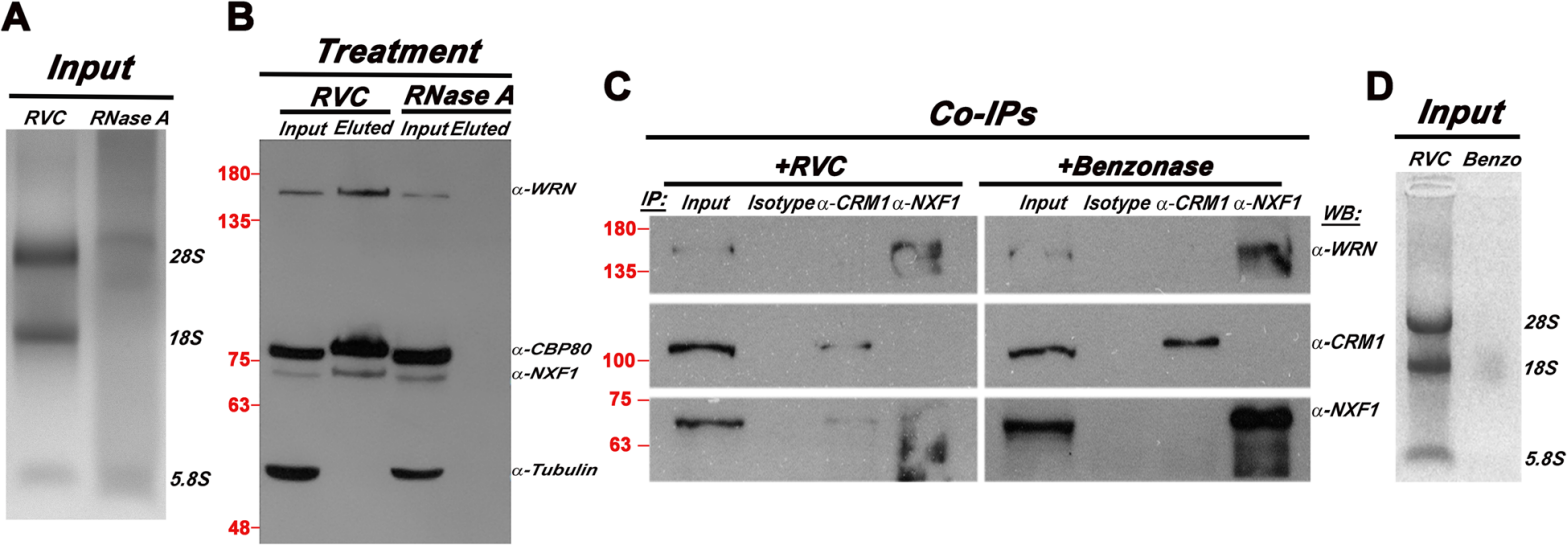
WRN interacts with the mRNA export receptor NXF1 and with mRNAs. **a** Whole-cell extracts were treated with RVC or RNase A (see Supplemental Information) and analyzed by EtBr-stained agarose gel after incubation with the Oligo (dT)_25_ magnetic beads. **b** Pull down assay with Oligo (dT)_25_ magnetic beads using whole-cell lysates from HeLa cells. The captured mRNPs were resolved in a polyacrylamide gel and analyzed by Western blot analysis using the indicated antibodies; 1% of the input was loaded in the first lane. CBP80 and tubulin were used as positive and negative controls, respectively. Molecular size markers (in KiloDaltons) are shown. This is a representative pull down of three independent experiments. **c** Co-IPs assays in HeLa whole-cell extract treated with RVC or Benzonase (see Supplemental Information for details) were performed using the indicated antibodies. The eluted immunocomplexes were analyzed by Western blots using the indicated antibodies. **d** EtBr-formaldehyde-agarose gels showing rRNA status after the treatment with RVC or Benzonase. This experiment was performed three times with identical results. Molecular size markers (in KiloDaltons) are shown

Since the mRNA export receptors NXF1 and CRM1 play key roles in nuclear export of RNA, we reasoned that WRN might aid these nuclear receptors during the export of mRNPs by directly interacting with mRNA and/or the export receptor. To test this hypothesis, we first examined whether WRN associates with mRNAs using Oligo (dT) pull-down. For this purpose, we isolated whole cell extracts from HeLa cells and divided them into two aliquots. One aliquot was treated with RNase A (DNase-Free) and the other with Ribonucleoside Vanadyl Complex (RVC) to protect from endogenous ribonucleases. Each fraction was incubated with Oligo (dT) beads and after extensive washes the proteins were eluted from the beads, resolved on SDS-PAGE and analyzed by immunoblotting using antibodies against the nuclear export receptor NXF1, WRN and the RNA-binding protein CBP80 as a control. An antibody against tubulin was used as a negative control. Both NXF1 and the mRNA binding proteins CBP80, but not tubulin, were efficiently pulled down only in the extracts that were not treated with RNase A (Fig. 6a and b), confirming the specificity of the assay to capture RNA-binding proteins. Remarkably, WRN was detected in the Oligo (dT) bound material from the RVC treated extract, but absent from the pull-down of the RNase A treated sample, indicating that pull-down of WRN is dependent on the presence of undamaged mRNA (Fig. 6b). This finding demonstrates a novel link between WRN and mRNA. Next, we examined whether WRN binds directly to the export receptors. For this purpose, whole cell extracts from HeLa cells were subjected to co-immunoprecipitation (Co-IPs) assays using antibodies against NXF1 or CRM1. The results of this experiment show that WRN co-precipitates with NXF1 but not CRM1 (Fig. 6c). Significantly, this interaction is not mediated by RNA, since it is resistant to Benzonase, which degrades nucleic acids (Fig. 6d), suggesting a direct and specific protein-protein interaction between WRN and NXF1.

## Discussion

Here, we provide the first evidence that WRN plays a role in RNA metabolism by influencing mRNA nuclear export. WRN is a RecQ helicase that through a network of protein-protein interactions contributes to several nuclear processes, including telomere length homeostasis, DNA replication, and repair [61–71]. Our results provide evidence of an additional function for WRN in a nucleic acid transaction. Given that rapidly proliferating cancer cells have an increased requirement of *de-novo* protein synthesis, our findings provide clues on how WRN may impact the growth of normal and cancer cells differentially. In previous studies, we demonstrated that WRN influences the levels of metabolic proteins that are required for the production of reducing equivalent NADPH [25]. These changes are not the result of altered gene expression (Li, et al. 2014 and Supplementary Fig. 1A), but rather a consequence of reduced mRNA export. Upon WRN depletion, a decrease in the levels of NADPH disrupts the reductive biosynthetic pathways and weakens the capacity to regenerates GSH, which plays a key role in ROS detoxification [25, 72, 73] resulting in the accumulation of reactive oxygen species [25, 74]. These cellular alterations are especially important for the growth and survival of cancer cells. In this work we show that these changes are associated with a general attenuation in *de-novo* protein synthesis (Fig. 1), indicating that deregulation by WRN depletion is more widespread than we anticipated. However, our previous studies indicated that a compromised reductive biosynthetic pathway is a major contributor to cell dysfunction since supplementation with the antioxidant agent glutathione rescues the proliferation of WRN depleted HeLa cells [25]. Oxidative stress leads to the accumulation of stress granules and P-bodies, which sequester stalled mRNAs, translation initiation factors, and other RNA binding proteins [38, 75, 76], severely limiting translation. We therefore examined whether formation of stress granule might explain translational attenuation after WRN depletion in HeLa cells. We determined that within the first three days of WRN depletion, when attenuation of protein synthesis is observed, there is no detectable accumulation of SGs (Supplementary Fig. 1B and C), nor an increase in the DNA damage [77, 78] (Supplementary Fig. 1D and E).

The rates of protein synthesis in proliferating cancer cells depends on the availability of functional ribosomes, and many RPs have been found to be upregulated in human cancers compared with their normal counterparts [79]. Our analysis does not reveal a reduction in either RPs or rRNAs (Fig. 2). Importantly, the results of RPs in WRN depleted HeLa cells show a significant increasing in the levels of RPs we analyzed, suggesting the activation of a possible mechanism of compensation to overcome the reduced export of mRNA. We observed a similar effect on the mammalian target of rapamycin (mTOR) pathway, which is a major regulator of ribosomal biogenesis and protein synthesis. mTOR phosphorylates the ribosomal protein S6 kinase (p70S6K1) leading to their activation and phosphorylation of substrates that promote translation initiation [80, 81]. We determined that WRN depletion does not affect mTOR and p70S6K1 protein levels (Supplementary Fig. 3). However, phosphor-p70S6K1 is upregulated at days 1 through 4 of WRN depletion (Supplementary Fig. 3). Since this phosphorylation event is known to stimulate translation, a plausible explanation of this result is that these cells attempt to compensate for the decrease in mRNA export by enhancing protein synthesis in order to sustain their proliferation. Interestingly, at 5 days of WRN depletion both mTOR and p70S6K1 protein levels decrease, possibly indicative of a global deregulation of cellular processes due to the major decrease in the proliferative potential of these cells at this time point [25]. Clearly more studies are needed to gain a better understanding of the underlying molecular mechanisms leading to these changes.

Efficient mRNA export is important for the proliferation of cancer cells. Upregulation of mRNA export factors has been observed in many cancer cell types and reducing export factors levels by siRNA inhibits cancer cell proliferation and abrogates mRNA export [59, 82–87]. Export factors play a key role in the transport of mRNPs across the nuclear pore complex (NPC) for translation in the cytoplasm [33, 88–90]. It has been reported that WRN interacts with a subset of nuclear pore proteins [35, 48, 91] including NDC1, a transmembrane nucleoporin [48]. Although the significance of these interactions is unknown, these findings suggested that WRN may contribute to a process, such as mRNA export that involves transport through the nuclear pore. Thus, we asked whether reduced levels of *de-novo* protein synthesis in WRN depleted cells could be the result of altered mRNAs export. Using two distinct approaches, we observed a significant difference in the nucleocytoplasmic distribution of the mRNAs between WRN depleted and control cells (Figs. 3 and 4). Notably, the altered nucleocytoplasmic ratio of mRNAs in HeLa cells is in part reverted by re-expression of WRN (Fig. 3e and f), which reinforces the concept that WRN is required for efficient mRNA export.

To better understand the relationship between WRN and the mRNA export machinery, we asked whether alterations in the levels of components of mRNA maturation and export machinery could explain the nucleocytoplasmic alterations in the mRNA distribution upon WRN depletion. Our experiments do not show any decrease in components between WRN-depleted and control cells (Fig. 5), ruling out defects in pre-mRNA processing and mRNP formation. On the other hand, we detected interactions between WRN and mRNAs as well as with the RNA export factor NFX1 (Fig. 6), suggesting that these interactions contribute to the nucleocytoplasmic transport of mRNAs. WRN interacts with the export receptor NXF-1 but not with CRM1, indicating a strict specificity in the export of mRNA. The observation that many cancer cell types display higher levels of WRN [22, 23] may reflect an increase requirement of mRNA export in these cells, as it is the case for other export proteins [59, 83, 84, 86]. Notably, as reported by others [24, 92] a small fraction of WRN is detected in the cytosolic compartment (Fig. 2b, Fig. 3a and b, and Supplementary Fig. 2B). This behavior is reminiscent of nuclear proteins that shuttle between the nucleus and the cytoplasm such as CBP80, PABP or ALYREF (reviewed in Gama-Carvalho and Carmo-Fonseca [93]) and further studies are needed to gain insight on this process. Since depletion or inactivation of WRN rapidly elicits senescence or apoptosis in many cancer cell types [22, 25, 64, 94], our data suggest that the regulation of mRNA export by WRN is important to sustain the proliferative potential of these cells. Importantly, the differential sensitivity of normal and cancer cells to acute WRN depletion points to a vulnerability in cancer cells that renders them more susceptible to WRN inhibitors. Future studies will further explore this important point.

## Conclusions

The identification of a role for WRN in regulating mRNA export and protein output has potential implication in cancer therapy. We envision that this new finding could aid in the design of approaches that sensitize cancer cells against chemotherapeutic drugs or slow down their proliferation by reducing mRNA export.

## Methods

A detailed description of the procedures used in this work is found in the Supplemental Information.

### Cell lines and culture

HeLa cell (CLS Cat# 300194/p772_HeLa, RRID:CVCL_0030) were purchased from ATCC. HeLa cells harboring the inducible shRNA system for WRN silencing (shWRN) or the scrambled shRNA as control (shCTR) at passage 9 and Werner Syndrome (WS) patient-derived fibroblasts (Coriell Cell repository Cat# AG06814-J, RRID:CVCL_0579) and its derivative over expressing Flag-WRN both at passage 5, were maintaining in high glucose Dulbecco’s modified Eagle’s medium (DMEM) containing L-glutamine and without sodium pyruvate (BIOWEST L0102–500). The medium was supplement with 10% heat inactivated fetal bovine serum (FBS), 100 U/mL penicillin and 100 μg/mL streptomycin. Cells were maintained in a humidified incubator at 37 °C in the presence of 5% CO_2_.

### Western blot analysis

After 3 days of incubation with doxycycline, cells expressing shWRN or shCTR were lysed on ice for 1 h with Lysis Buffer containing Tris-HCl, pH 7.6 (25 mM); NaCl (420 mM); NP-40 (0.2%); EDTA (1 mM); Protease Inhibitor Cocktail (PIC; P8340, Sigma-Aldrich) (1/100 (v/v)) and freshly prepares PMSF (1 mM). For the experiments conducted to detect phosphorylation status of mTOR and p70S6K we use the same Lysis buffer without EDTA and supplemented with Sodium Fluoride (NaF; 1 mM); Sodium Orthovanadate (Na_3_VO; 1 mM) and β-Glycerophosphate (β-GP; 1 mM), as selective inhibitors of protein phosphoseryl and phosphothreonyl phosphatases, protein phosphotyrosyl phosphatases and protein serine-threonine phosphatase, respectively, Protease Inhibitor Cocktail (PIC; P8340, Sigma-Aldrich) (1/100 (v/v)) and freshly prepared PMSF (1 mM). The extracts were clarified at 13,000 x g for 30 min at 4 °C and protein concentration was measured by Bradford Assay (Sigma-Aldrich B6916). Samples were resolved in 8–10% PAA gel using Opti-protein XL marker (G266 abm) as molecular size marker. The gels were then electro-transferred to a nitrocellulose membrane. One hour after blocking at room temp (RT) with blocking solution (5% non-fat milk in 1X Phosphate buffer saline containing 1% Tween-20 (1X PBS-T)), the membranes were incubated overnight with the respective primary antibodies prepared in blocking reagent at 4 °C in a roller mixer (a detailed list of the used antibody in this publication can be found in Supplementary Table 1). After several washes, the membranes were then incubated with the respective HRP-conjugated secondary antibodies (Supplementary Table 1) for 1 h at RT. HRP chemiluminescence was detected using ECL Western blotting Substrate (Thermo Fisher Scientific) and signals captured by X-ray radiography or ChemiDoc with a CCD camera from Bio-Rad. Gel bands were quantified using NIH ImageJ1.51j8 (ImageJ, RRID:SCR_003070, [95]).

### Inducible Tet-ON/Tet-OFF lentiviral shRNA vectors

HeLa cells were transduced with lentiviruses for the conditional expression of small hairpin (sh) targeting WRN (shWRN) or a scrambled control (shCTR) as described in our previous work [25]. For shRNA induction, the cells were treated with doxycycline (dox) (1.5 μg/mL) for the indicated number of days. In our studies we typically use a three-day dox treatment, which results in at least 80% reduction in WRN. The levels of WRN in control and WRN knockdown cells were estimated by Western blotting using antibodies against WRN and α-tubulin or actin as loading controls. Bands were quantified using the NIH ImageJ software.

### Metabolic labeling using ^35^S-met/cys

For the analysis of *de-novo* protein synthesis, we collect equal number of radiolabeled cells and prepared two aliquots with equal volumes of shCTR and shWRN cells extracts for analyses by 10% PAA gels. One of the PAA gels was used for Coomassie Brilliant Blue (CBB) staining (loading control) while the second was used for ^35^S-met/cys measurement. Both gels were vacuum-dried, exposed to a phosphoscreen and developed using a Pharos FX molecular imager (BIO-RAD). The data were plotted using Graph Pad Prism (GraphPad Prism, RRID:SCR_002798) and the significance determine by Student’s *t*-test of three independent samples.

### Immunoprecipitation of ^35^S-met/cys labeled proteins

Extracts derived from ^35^S-met/cys labeled cells samples were subjected to immunoprecipitation assay. To this end, we used the same concentration of specific antibodies against G6PD, Ku70 and Tubulin. Reactions were incubated overnight at 4 °C in a roller and the immunocomplexes were captured using Protein A/G PLUS-Agarose (Santa Cruz Biotechnology, sc-2003,) following the manufacturer’s instructions. The immunoprecipitated complexes were resolved in 10% PAA and stained with CBB followed by vacuum drying. Lastly, the gels were scanned to visualize IgG and then exposed to Phosphoscreen and imaged using a Pharos FX molecular imager (BIO-RAD).

### Nuclear-cytoplasmic fractionation and preparation of Polysome enriched fraction (PEF)

This procedure was performed in two steps. Briefly, for the preparation of nuclear and cytoplasmic fractions, the shCTR and shWRN dox-treated cells and WS-derived fibroblasts were lysates using hypotonic buffer containing NP-40 (0.1%). The cell extracts were them centrifuged at low RPM to separated nuclei from the rest of the cell components. The supernatants were centrifuged at high RPM and the newly resulted supernatants were separated and termed cytoplasmic fraction. The nuclear fraction from above was extracted in hypotonic buffer supplemented with NaCl (420 mM). The extracts were collected by high RPM centrifugation. Equal volumes of each fractions were resolved by SDS-PAGE and probed with nuclear and cytoplasmic markers. For the preparation of the polysome-enriched fraction, the cytoplasmic fractions obtained as described above were loaded on a sucrose cushion and subjected to ultra-centrifugation. The pellets were quickly rinsed once in hypotonic buffer and resuspended in 2X loading buffer for proteins analysis or directly extracted by TRIzol for RNA extraction.

### Gene expression analysis by RT-qPCR

We used TRIzol for all RNA extraction process following the manufacturer’s instructions. For RT-qPCR analysis, 2 μg RNAs was subjected to a step-by-step process to generates the cDNA. We used18S rRNA or GAPDH as internal controls. Samples were analyzed in triplicates (of three biological replicates), and quantification was performed by comparing the values obtained at the fractional number of a cycle at which the amount of amplified target reaches a fixed (Ct) threshold. For evaluation of the differences in means between the two groups in the analysis of the rRNA abundance and for the alteration in ribosomal proteins (RP) genes, we used two-way ANOVA followed by Sidak’s multiple comparisons test, since Sidak’s theorem provides a more rigorous multiple comparison method by adjusting the significance level [96]. Alike, for the nuclear and cytoplasmic fractionations assays followed by qPCR analysis in shCTR and shWRN cells, we used two-way ANOVA followed by Sidak’s multiple comparisons test. All these statistical analyses were performed using GraphPad Prism Version 8.3.0 for Windows GraphPad Software, San Diego, California USA (GraphPad Prism, RRID:SCR_002798) Version 8.4.2. Experiments for the analysis of WS patient-derived fibroblast and recovery of WRN protein in HeLa cells were conducted twice, thus we calculated the mean ± SEM of two biological replicates without statistical analysis.

### Immunofluorescence and stress granules formation (SG) assay

To induce SGs formation in shCTR and shWRN cells, the cells were treated with sodium arsenate after dox treatment. After fixing, the cells were permeabilized and blocked before labeling with the appropriate primary and secondary antibodies using Phalloidin and DAPI as counterstain. The samples were mounted using HardSet Antifade Mounting Medium and curated for 24 h in the dark at RT before visualized. The samples were visualized using a confocal laser scanning microscope (Nikon).

### mRNA fluorescence in situ hybridization (FISH)

RNA FISH was performed as previously described in Viphakone, N. et al., 2012 [55] with some modifications. A completed detailed protocol is found in the Supplemental Information. For the FISH analysis we used two-tailed unpaired *t* test, using GraphPad Prism Version 8.3.0 for Windows GraphPad Software, San Diego, California USA.

### Co-Immunoprecipitation assays

We used whole cell lysates for co-immunoprecipitation (Co-IP) assays. The clarified extracts were incubated with the antibodies indicated in the main text and Supplementary Table 1. The immunocomplexes were recovered using equilibrated Protein G Magnetic Beads (NEB S1430S) and eluates were resolved by SDS-PAGE and probed with the respective antibodies and detected by ECL reaction using X-ray films.

### mRNA pull down assay

HeLa cells at 75% confluency were washed with ice-cold 1X PBS twice, scraped off from the plates and pelleted by centrifugation at 1500 x g for 5 min at 4 °C. The cell pellets were resuspended, divided into two equal volumes in RNase/DNase-free pre-chilled 1.5 mL tubes and pelleted again. Cells were lysate for 1.5 h on ice with Lysis Buffer (see Supplemental Information) for 30 min on ice, the crude extracts were clarified at 15,000 x g for 30 min at 4 °C and supernatants transferred to a new RNase/DNase-free ice-chilled tube. An aliquot was used to assay RNA integrity. mRNA was pulled down using Oligo (dT) Magnetic Beads for 2 h at 4 °C in a roller mixer and an aliquot was used again to assay RNA integrity. After extensive washes, the bound material was eluted by adding loading buffer and heating at 95 °C for 5 min. The eluates were resolved by SDS-PAGE and subjected to Western blot analysis.

### Analysis of mTOR and p70S6K1

shWRN and control HeLa cells were incubated for five days in the presence of dox. During the time course, fresh media with doxycycline was added to the cell each day. At each time point, the cells were washed with ice-cold 1X PBS twice, scraped off from the plates and pelleted by centrifugation at 1500 x g for 5 min at 4 °C. The cells were then lysed on ice for 1 h with Lysis Buffer. After clarification by centrifugation, the protein concentration was measured by Bradford Assay and samples were resolved on 8% PAA gels. After electro-transfer, the membranes were probed using the indicated antibody.

## Supplementary information

**Additional file 1.** Original images. Original uncropped images used to generate the figures shown in the manuscript.

**Additional file 2: Supplementary Fig. 1.** RT-qPCR analysis of metabolic genes, stress granules formation and oxidative DNA damage. **(A)** Quantitative RT-qPCR analysis of G6PD and IDH1 using B2M as the internal control were performed as described in [25]. The results of four biological replicates are plotted using GraphPad Prism. Two-way ANOVA followed by Sidak’s multiple comparisons test was used to calculate the significance. The error bars represent the mean ± SEM (*n* = 4). ** *p* value < 0.005; ns, no significant differences. **(B)** Western blot analysis showing WRN depletion in HeLa cells that were used for the SG formation experiment. Molecular size markers (in KiloDaltons) are shown. **(C)** WRN-depleted and control HeLa cells were seeded in an 8-well chamber slide. After fixation and permeabilization, the cells were incubated with the respective antibodies (see Supplemental Information and Supplementary Table 1) and counterstain solutions. The samples were analyzed by immunofluorescence using confocal microscopy. Treatment with 3 mM sodium arsenite for 2 h was used to induce stress granule formation in both cell lines (Scale bar = 50 μm). Expanded boxed regions are shown on the right (Scale bar = 20 μm). **(D)** Representative Western blot analysis showing reduced levels of WRN after dox treatment. (Right panel) bands quantification. **(E)** The extracts were assayed for protein content using the Bradford method and same amount of proteins were loaded on a polyacrylamide gel. The samples were probed for phosphor-γ-H2AX and tubulin was used as the loading control. Two different amounts of extracts were used to better visualize potential changes in phosphor-γ-H2AX. (Right panel) bands quantification. Molecular size markers (in KiloDaltons) are shown.

**Additional file 3: Supplementary Fig. 2.** Nuclear/cytoplasmic fractionation and polysomes purification. Differential centrifugation followed by ultracentrifugation on a 30% sucrose cushion bed was used to generate five fractions. **(A)** A schematic representation of the procedure is shown. **(B)** Equal volumes of each fraction were loaded onto each lane. No cross contamination was observed in the fractions using PARP1 (nuclear), G6PD (Cytosol), Cox6b1 (mitochondria), RPL7a (ribosomes) and actin. The lack of detection of any of these markers in the Polysome Enriched Fraction (PEF) indicates the purity of this fraction which was used for the analysis of the 5.8S, 18S and 28S RNA by qPCR (Fig. 2c). This experiment was performed several times with identical results.

**Additional file 4: Supplementary Fig. 3.** Analysis of mTOR and its downstream target P70S6K1 in WRN depleted and control HeLa cells. Western blot analysis of the dox time course experiment in shWRN and shCTR HeLa cells. Whole cell extracts were resolved by SDS-PAGE and immunoblotted against the indicated antibodies. The same extracts were loaded in three different gels and actin was used as a control in each blot. Bands intensities were quantitated using Image J and plotted into a graph.

**Additional file 5: Supplementary Table 1.** List of the primary and secondary antibodies used in this study.

**Additional file 6 Supplementary Table 2*.*** List of the primers for qPCR used in this study.

**Additional file 7.** Supplemental Information.

## Data Availability

All data generated or analysed during this study are included in this published article and its supplementary information files.

## Abbreviations

WS: Werner Syndrome
WRN: Werner Syndrome Protein
ROS: Reactive Oxygen Species
G6PD: Glucose-6-phosphate dehydrogenase
IDH1: Isocitrate dehydrogenase
hnRNAs: Heterogeneous RNA
mRNA: messenger RNA
rRNA: Ribosomal RNA
mRNP: messenger Ribonucleoprotein
snRNAs: Small nuclear RNA
NPC: Nuclear Pore Complex
NE: Nuclear Envelope
Nups: Nucleoporin
TPR: Translocated promoter region protein
NXF1: Nuclear Export Factor 1
CRM1: Chromosome Maintenance Protein 1
TREX-1: TRanscription-EXport complex 1
UAP56: ATP dependent RNA helicase
ALYREF: Aly/REF Export Factor also known as THOC4
THO: subcomplex of the TRanscription-EXport complex 1
THOC1: THO complex subunit 1
THOC2: THO complex subunit 2
THOC3: THO complex subunit 3
THOC5: THO complex subunit 5
THOC6: THO complex subunit 6
THOC7: THO complex subunit 7
CIP29: Cytokine-Induced Protein
CBP80: Cap-Binding Protein 80, also known as NCBP1
GANP: Germinal center–Associated Nuclear Protein
eIF4E: Eukaryotic translation initiation factor 4E
WHIP1: Werner Helicase Interacting Protein 1
NDC1: Nuclear Division Cycle 1 a transmembrane nucleoporin
Ku70: ATP-dependent DNA helicase 2 subunit KU70
SGs: Stress Granules
TIAR: TIA-1-related protein
γ-H2AX: Phosphorylated Histone 2AX
FISH: Fluorescent In Situ Hybridizations
Poly (A)^+^ RNA: Poly Adenylated RNA or mRNA
RVC: Ribonucleoside Vanadyl Complex
POM: Pore Membrane Protein
shWRN: small hairpin complementary oligonucleotides of WRN gene
shCTR: Scramble small hairpin RNA (shRNA)
F-WRN: Human WS derived fibroblasts carrying Flag-WRN
SDS-PAGE: Polyacrylamide-gel electrophoresis
RT-qPCR/qPCR: Quantitative PCR
Co-IP: Co-Immunoprecipitation
