# Supplementary material for: WRN modulates translation by influencing nuclear mRNA export in HeLa cancer cells
Source: BMC Mol Cell Biol. 2020 Oct 14;21:71. doi: 10.1186/s12860-020-00315-9 (PMC7557079; doi:10.1186/s12860-020-00315-9)
Supplement: Supplementary file 1 — Additional file 1. Original images. Original uncropped images used to generate the figures shown in the manuscript. [file 12860_2020_315_MOESM1_ESM.pdf]

WRN modulates translation by influencing nuclear mRNA export in HeLa cancer cells

Juan Manuel Iglesias-Pedraz; Diego Matia Fossatti Jara; Valeria del Carmen Valle-Riestra Felice; Sergio Rafael Cruz Visalaya; Jose Antonio Ayala Felix and Lucio Comai.

Additional file 1
(Original images)

^{35}S-met labeling

1A

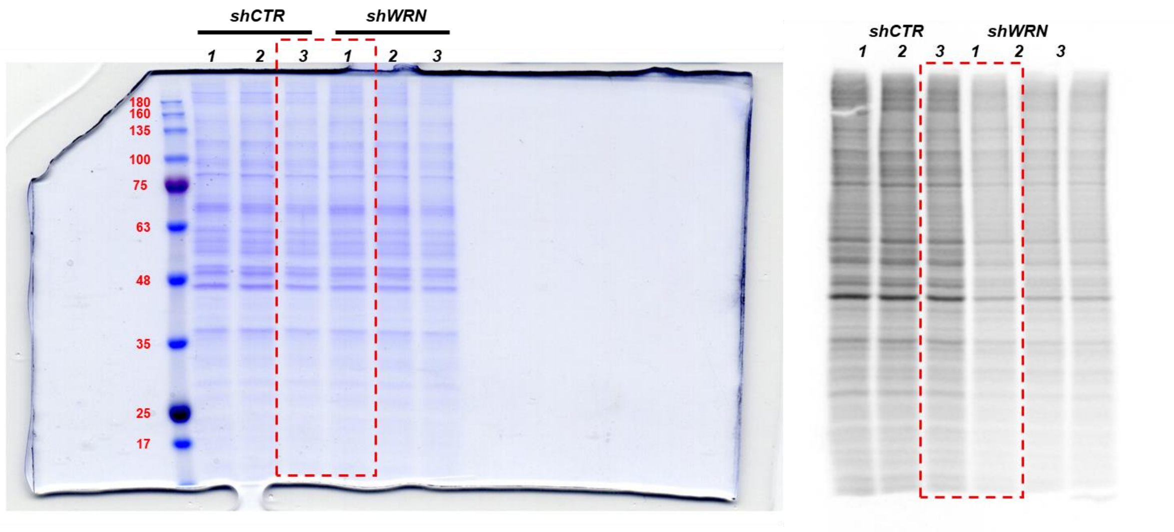

Original file 1. Original Coomassie stained PAGE and the corresponding phosphor screen image to detect ^{35}S-met/cys shows in **Figure 1A**. The numbers denote three replicates for each groups of cells. Molecular weight marker sizes are shown. The red dotted-line squares denote the cropped areas used for generate the Figure.

Western blot for ^{35}S-met labeling

1B

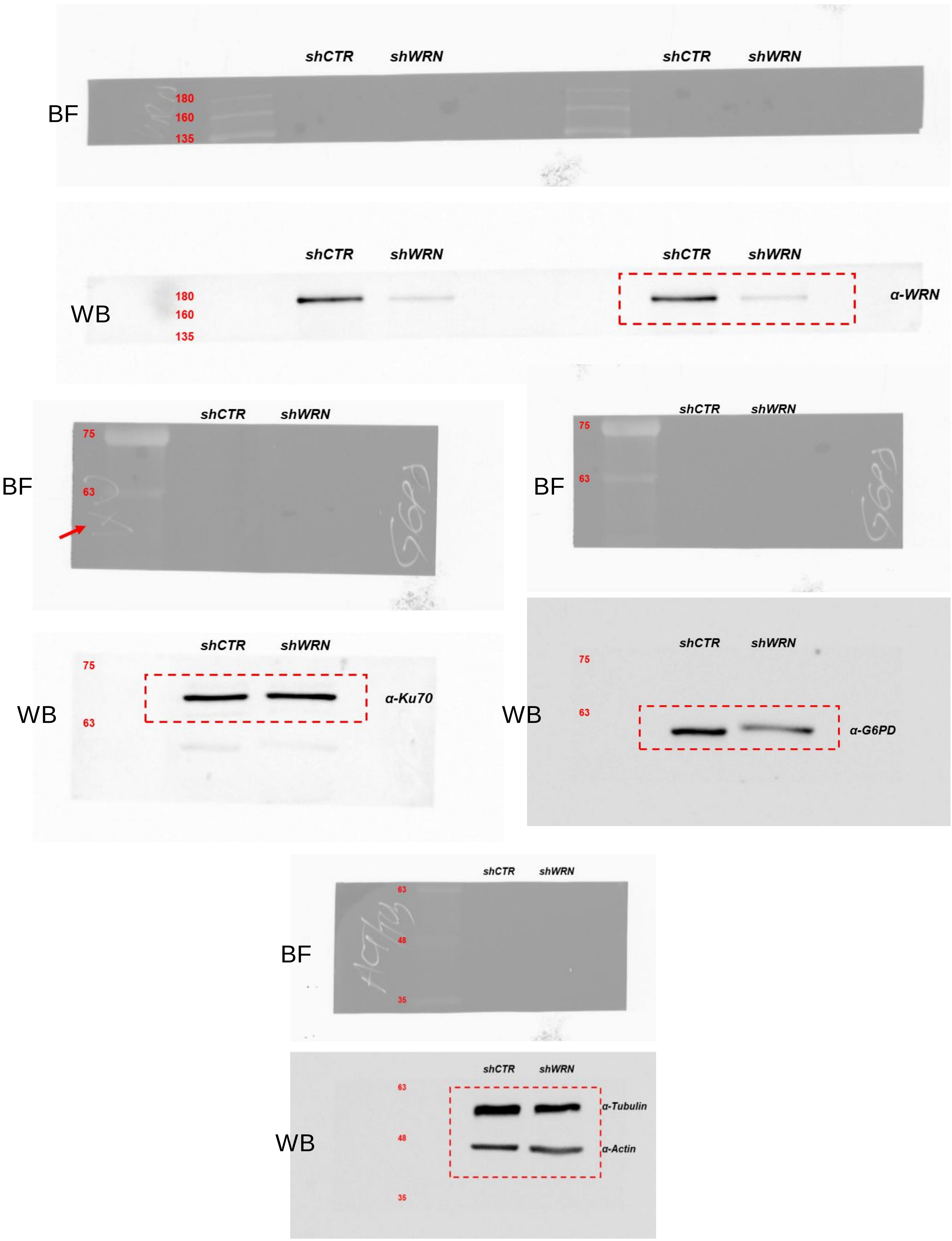

***Original file 2*.** Original membranes and WB for **Figure 1B**. The HRP chemiluminescent signals was captured by ChemiDoc with a CCD camera from Bio-Rad. The red arrow shows the antibody used in this membrane (Ku70). Molecular weight marker sizes are shown. The red dotted-line squares denote the cropped areas used to generate the Figure. BF, Bright field; WB, Western blot.

^{35}S-met labeling Immunoprecipitation

1C

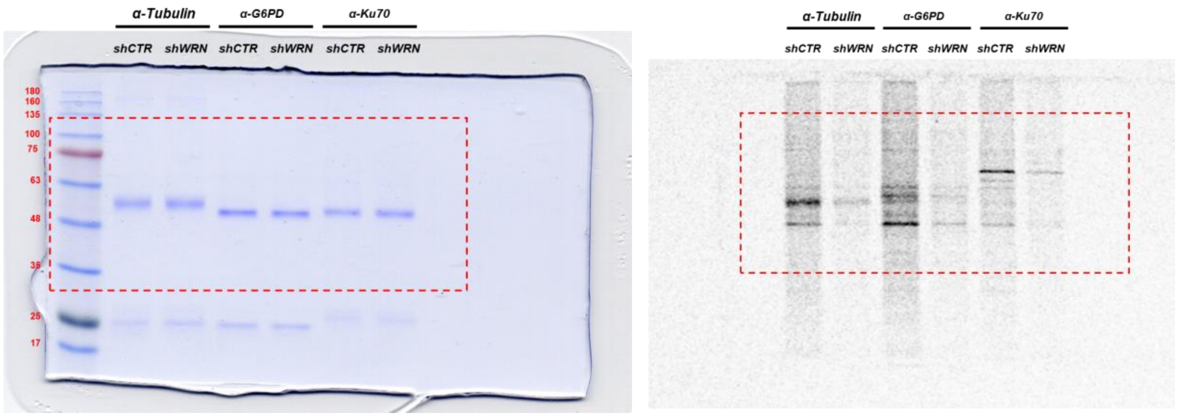

Original file 3. Original PAA gel and phosphor screen exposure show in **Figure 1C**. Molecular weight marker sizes are shown. The red dotted-line squares denote the cropped area used for generate the Figure.

Subcellular fractionation and Ribosomal protein analysis

2A

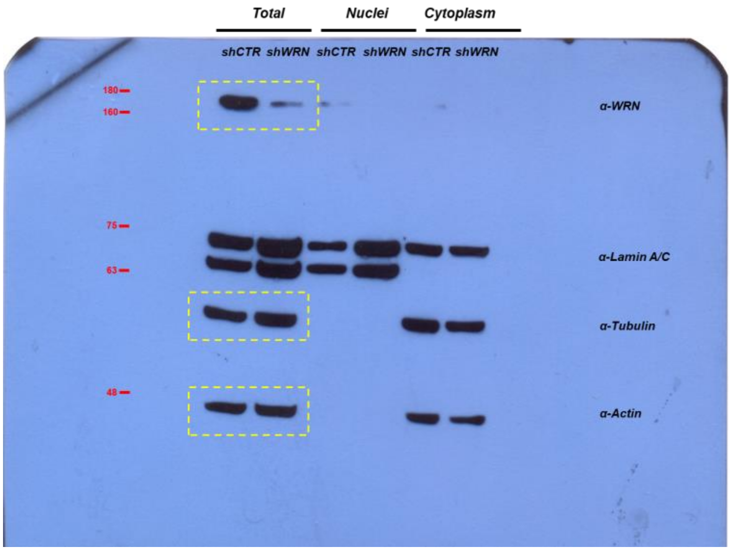

2D

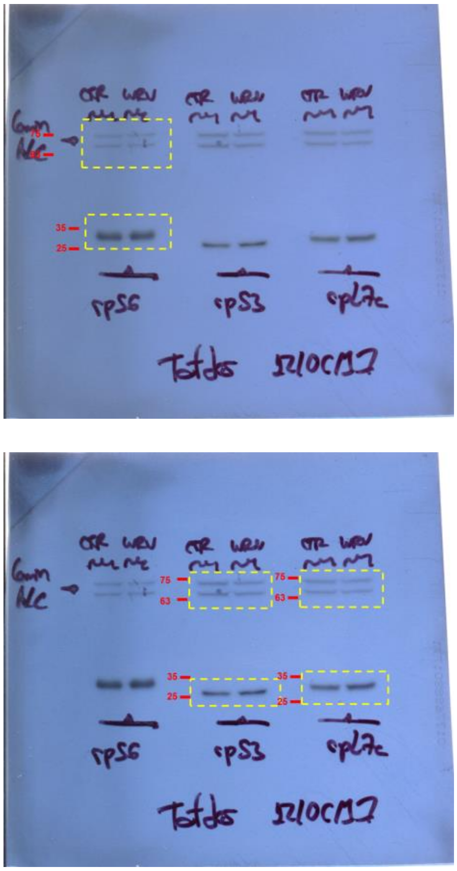

2B

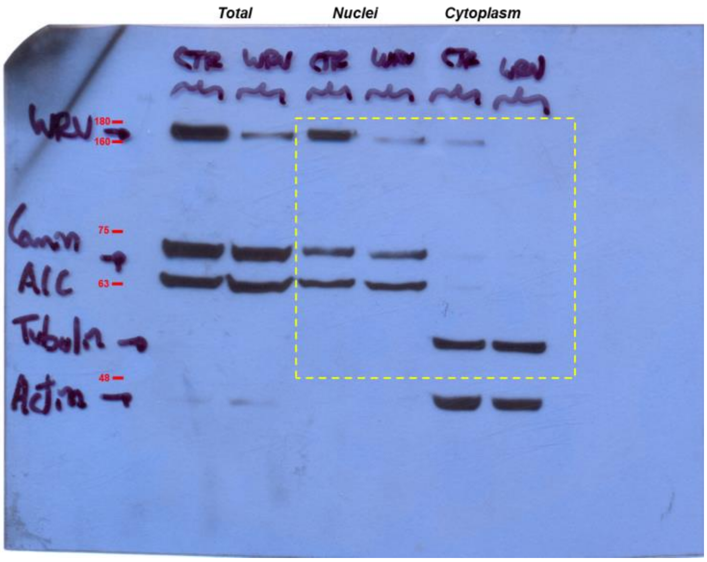

Original file 4. Original Western blots for **Figures 2A, 2B and 2D**. Molecular weight markers sizes are shown. The HRP chemiluminescent signals was captured by using X-ray film. The yellow dotted-line squares denote the cropped areas used to generate the Figure.

Subcellular fractionation in HeLa shCTR and shWRN cells

3A

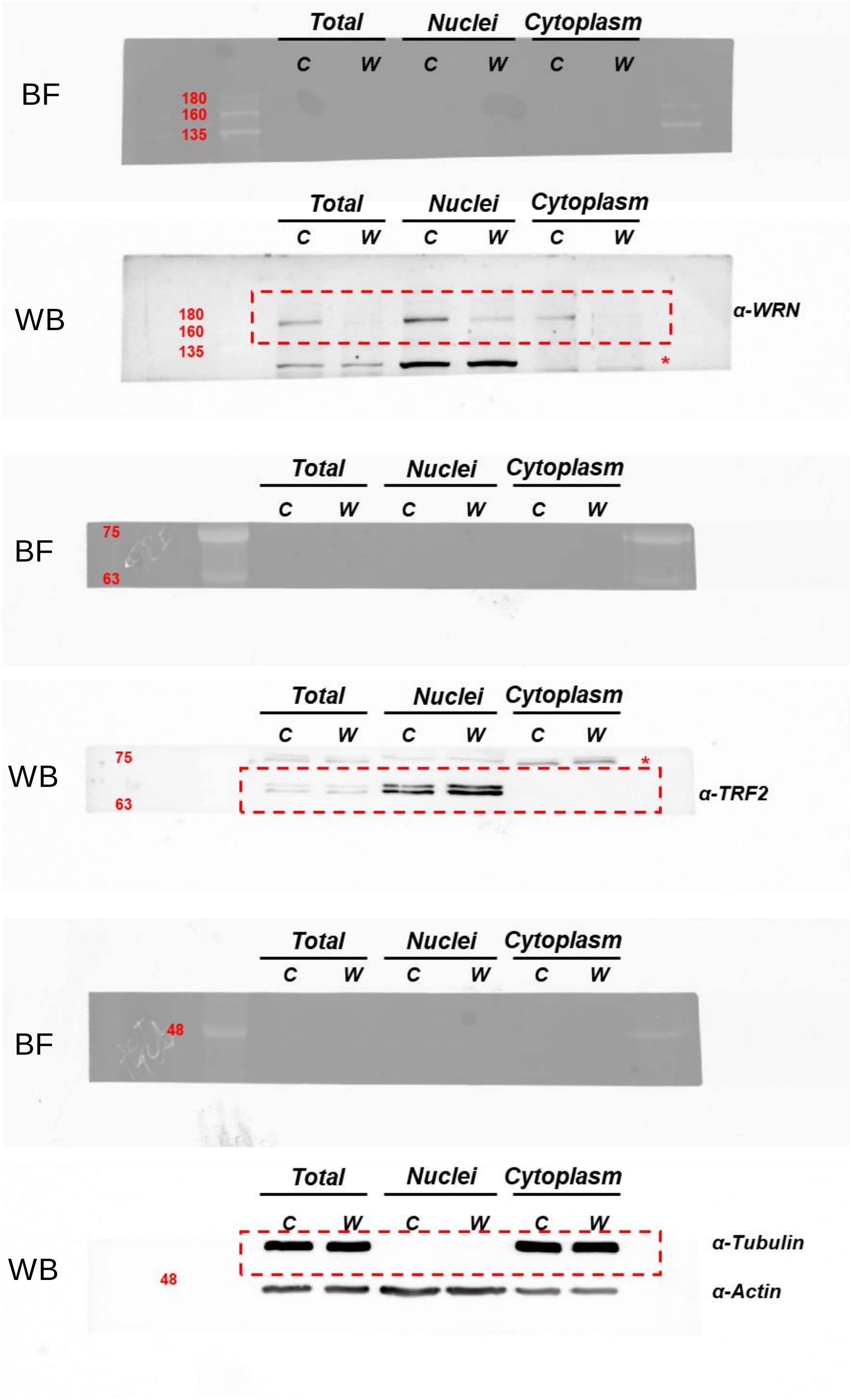

Original file 5. Original Western blot for **Figure 3A**. Molecular weight markers are shown. *****The asterisk denote unspecific cross-reactivity. The HRP chemiluminescent signals were captured by ChemiDoc with a CCD camera from Bio-Rad. The red dotted-line squares denote the cropped areas used to generate the Figure. C, shCTR and W, shWRN. BF, Bright field; WB, Western blot.

Subcellular fractionation in WS fibroblasts

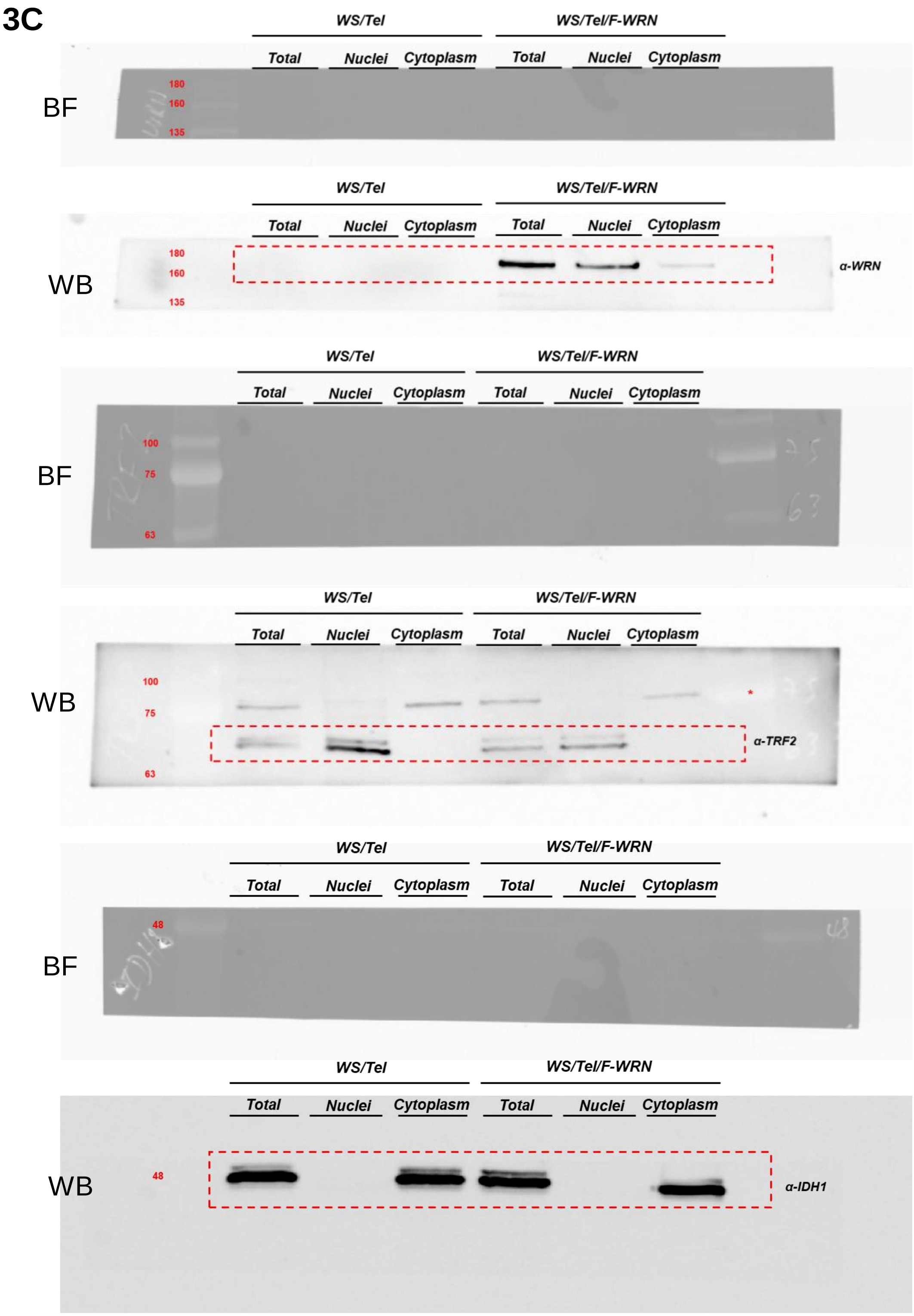

Original file 6. Original images of **Figure 3C**. *****The asterisk denotes unspecific cross-reactivity. Molecular weight markers are shown. The HRP chemiluminescent signals were captured by ChemiDoc with a CCD camera from Bio-Rad. The red dotted-line squares denote the cropped areas used to generate the Figure. BF, Bright field; WB, Western blot.

Western blot of WRN recovery in HeLa cells

3E

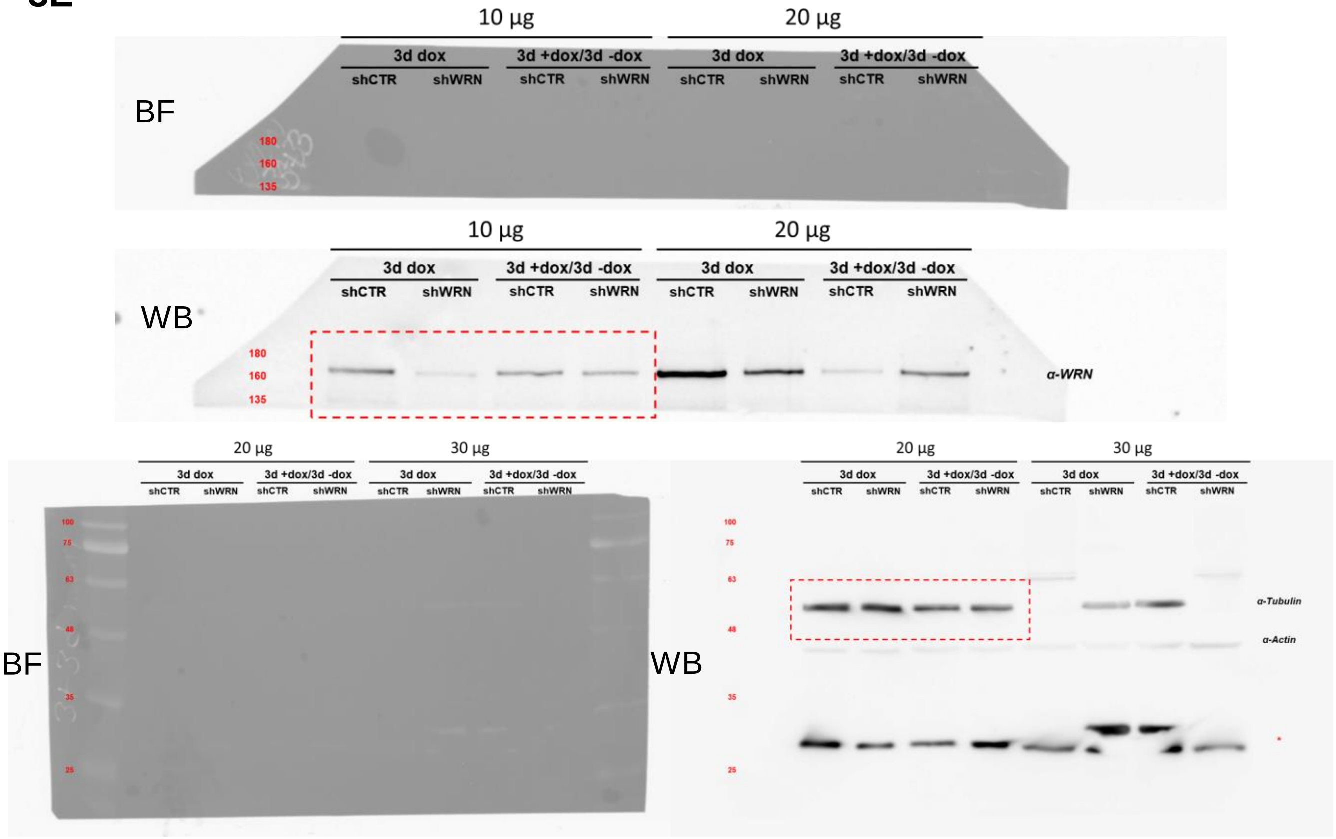

Original file 17. Original images of the **Supplementary Figure 3E**. Molecular weight markers are shown. The HRP chemiluminescent signals were captured by ChemiDoc with a CCD camera from Bio-Rad. The red dotted-line squares denote the cropped areas used to generate the Figure. BF, Bright field; WB, Western blot.

Western blot of WRN depletion in HeLa cell for FISH analysis

4A

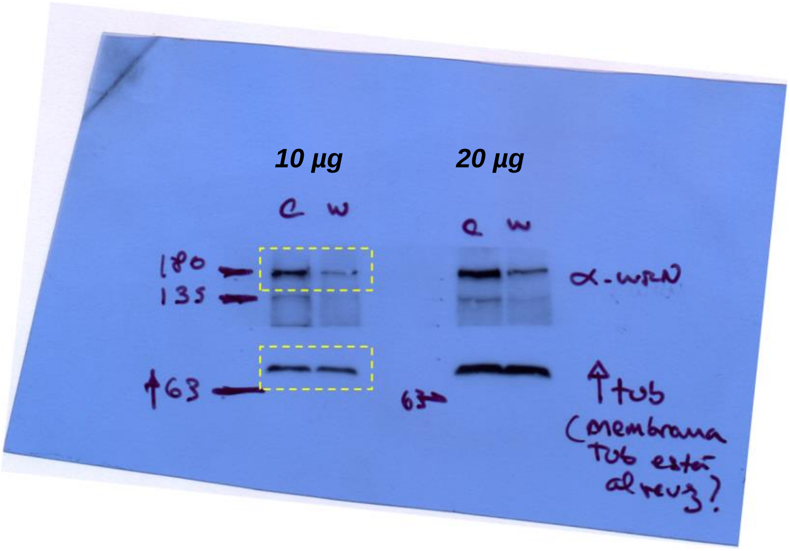

Original file 7. Original immunoblot of the **Figure 4A**. The molecular wight marker are indicated. The HRP chemiluminescent signals was captured by using X-ray film. C, shCTR; W, shWRN. The yellow dotted-line squares denote the cropped areas used to generate the Figure.

Western blot of RNA export receptors in HeLa cells

5A

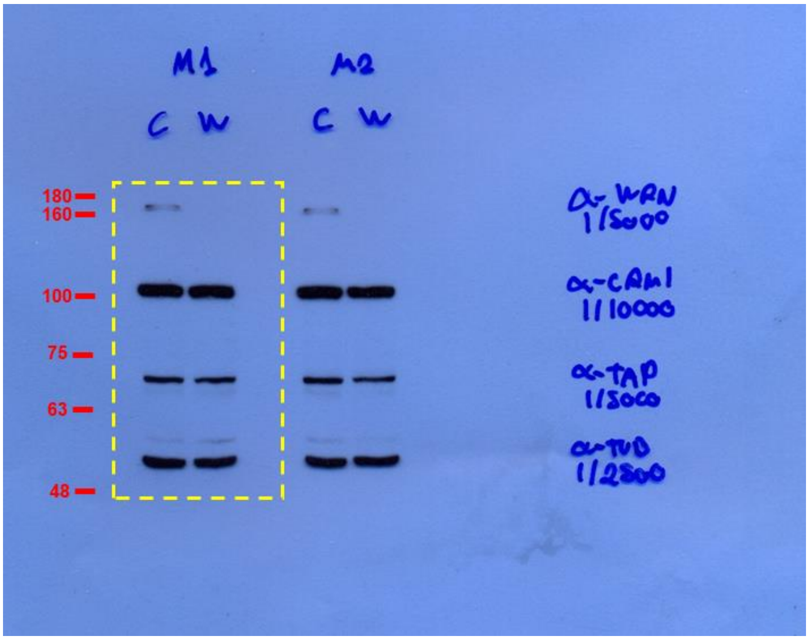

Original file 8. Original immunoblot of **Figure 5A**. The HRP chemiluminescent signals were captured by using X-ray film. M1 and M2 are replicate samples. C, shCTR and W, shWRN. TAP = NXF1. The yellow dotted-line square denotes the cropped area used to generate the Figure.

Western blot of RNA export factors in HeLa cells

5B

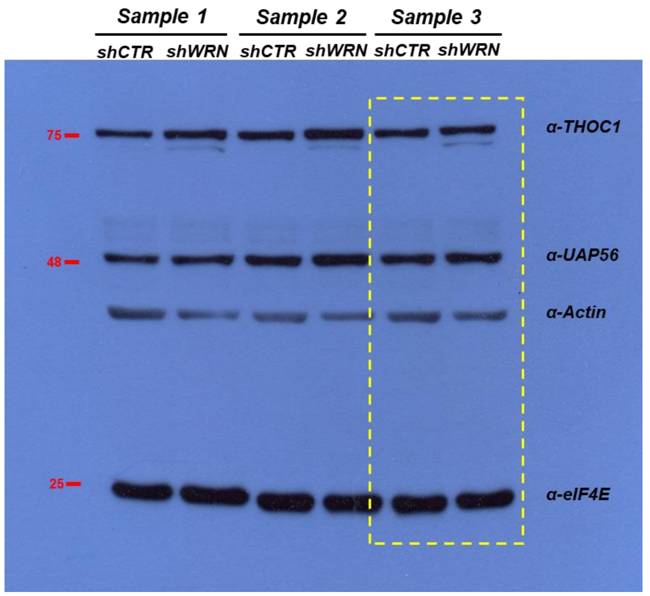

5C

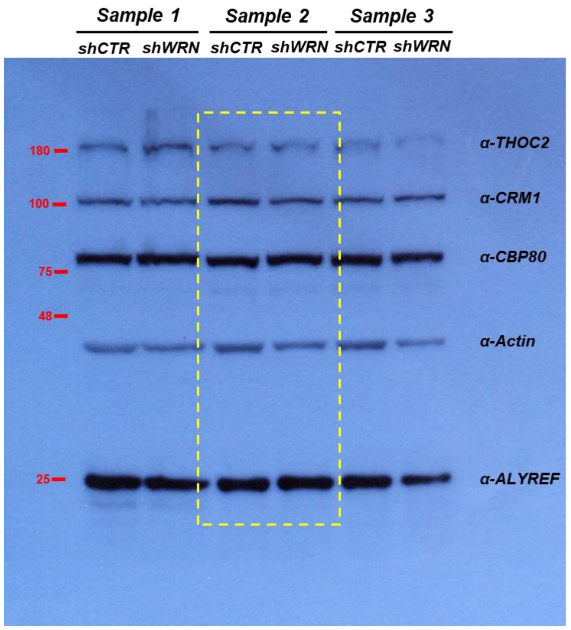

5D

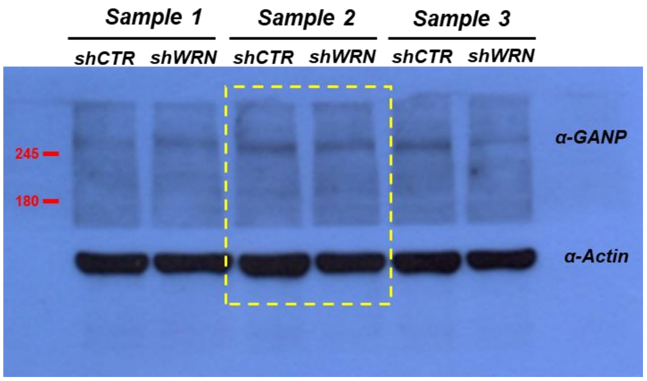

Original file 18. Original images of **Supplementary Figures 5B, 5C and 5D**. The HRP chemiluminescent signals were captured by using X-ray film. The yellow dotted-line squares denote the cropped areas used to generate the Supplementary Figure. Molecular weight markers are shown.

RNA integrity after RNase A treatment in HeLa cells

6A

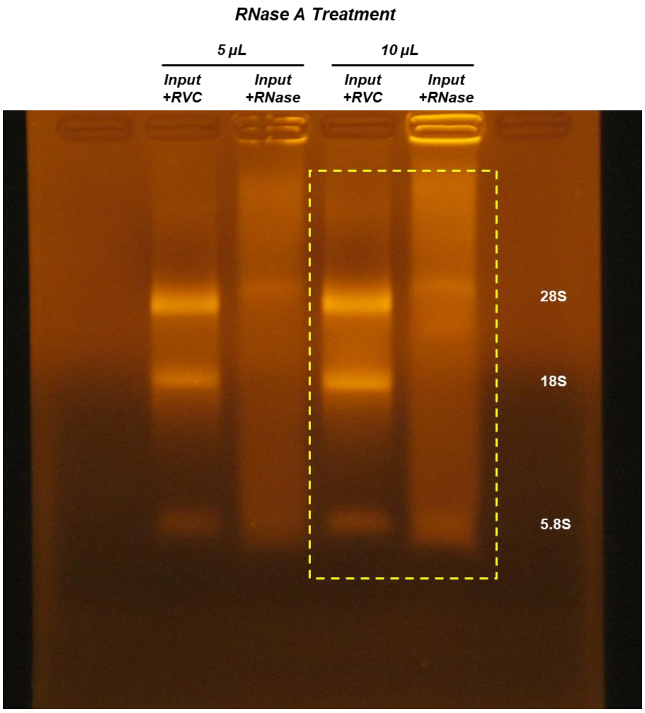

Original file 9. Original formaldehyde-agarose gel of **Figure 6A**. The gel was stained with Ethidium Bromide and exposed to UV light. The yellow dotted-line square denotes the cropped area used to generate the Figure.

Western blot of Oligo(dT) pull down assay in HeLa cells

6B

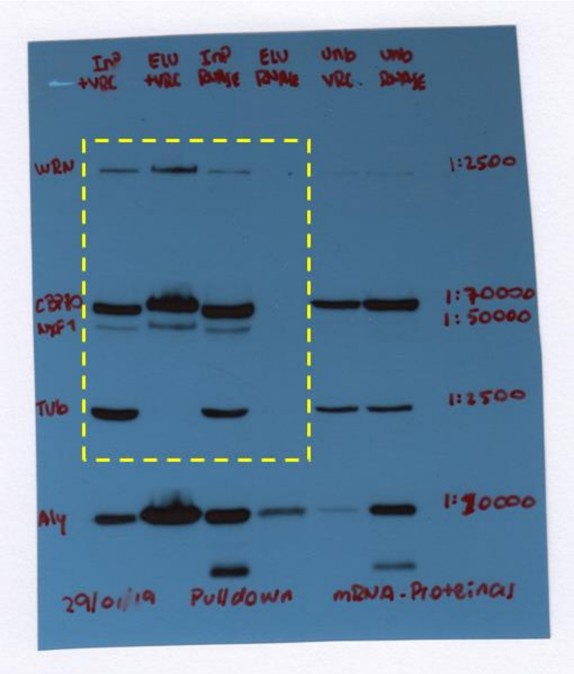

Original file 10. Original Western blot of **Figure 6B**. Inp:Input; Elu: Eluted fraction; Unb: unbound fraction. The HRP chemiluminescent signals were captured by using X-ray film. The yellow dotted-line square denotes the cropped area used to generate the Figure.

Western blot of Co-immunoprecipitation assay in HeLa cells

6C

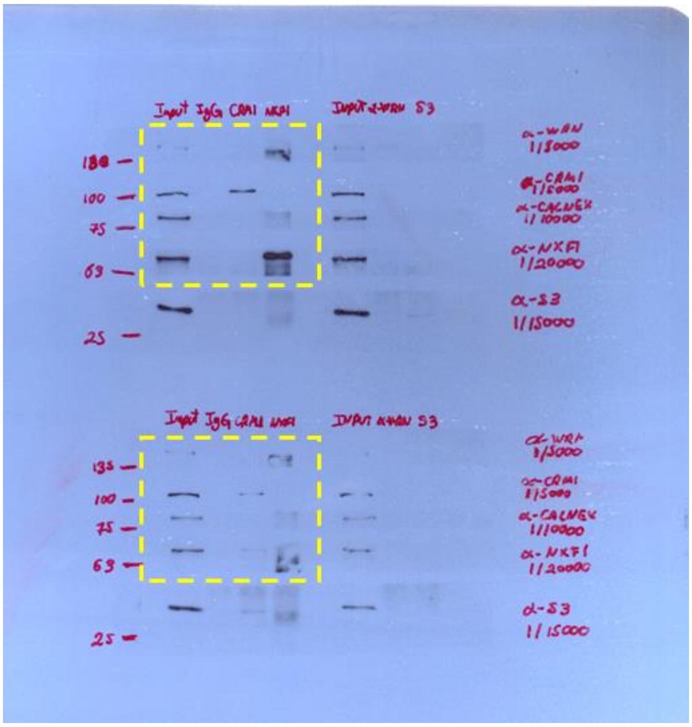

Original file 11 Original Western blot of **Figure 6C**. The HRP chemiluminescent signals was captured by using X-ray film. The yellow dotted-line squares denote the cropped areas used to generate the Figure.

RNA integrity after Benzonase treatment in HeLa cells

6D

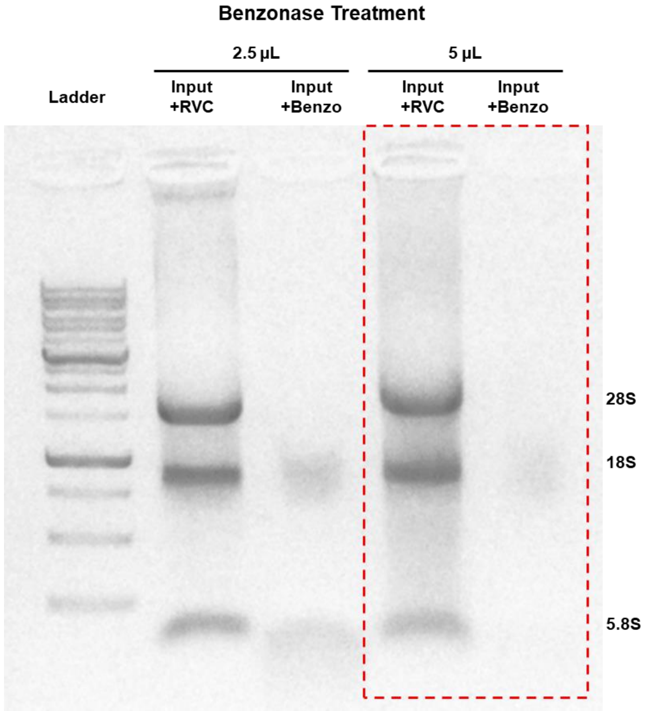

Original file 12 Original formaldehyde-agarose gel of **Figure 6D**. The ladder is a DNA ladder that was used as reference. The gel was stained with Ethidium bromide and exposed to a UV light. The red dotted-line square denotes the cropped area used for generate the Figure.

Western blot of WRN depletion in HeLa cell for SGs assay

Supp. Fig 1B

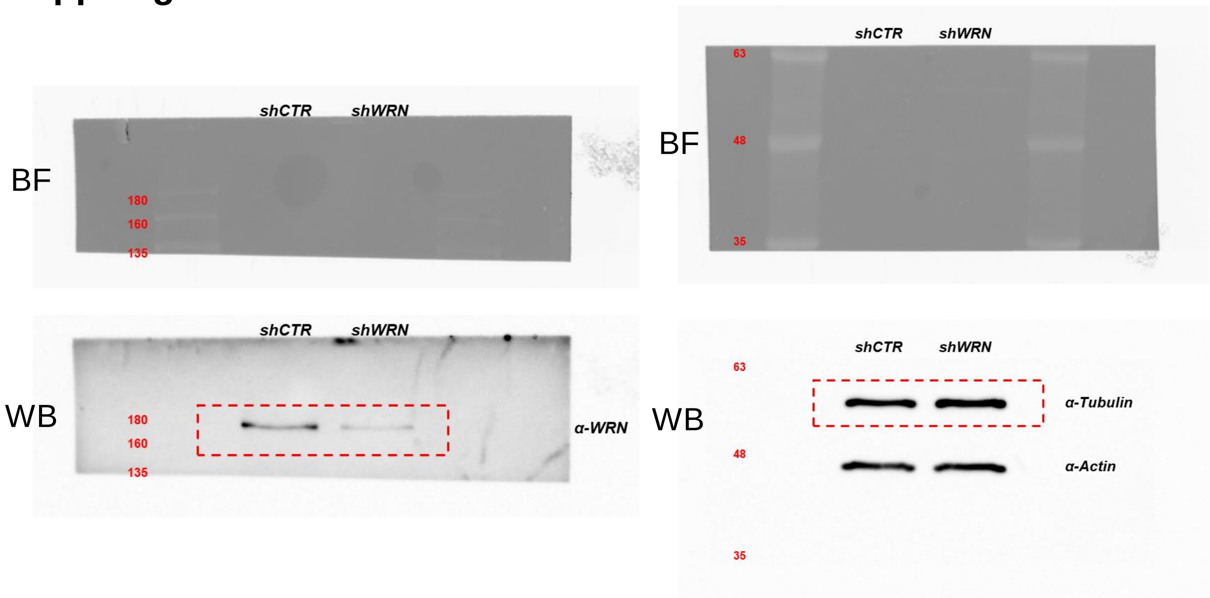

Original file 13 Original WB of **Supplementary Figure 1B.** Molecular weight marker are shown. The HRP chemiluminescent signals was captured by ChemiDoc with a CCD camera from Bio-Rad. The red dotted-line squares denote the cropped areas used to generate the Supplementary Figure. Molecular weight marker are shown. BF, Bright field; WB, Western blot.

Western blot of phosphor-γ-H2AX marker in WRN-depleted HeLa cells

Supp. Fig 1D

Supp. Fig 1E

Short exposure **Long exposure**

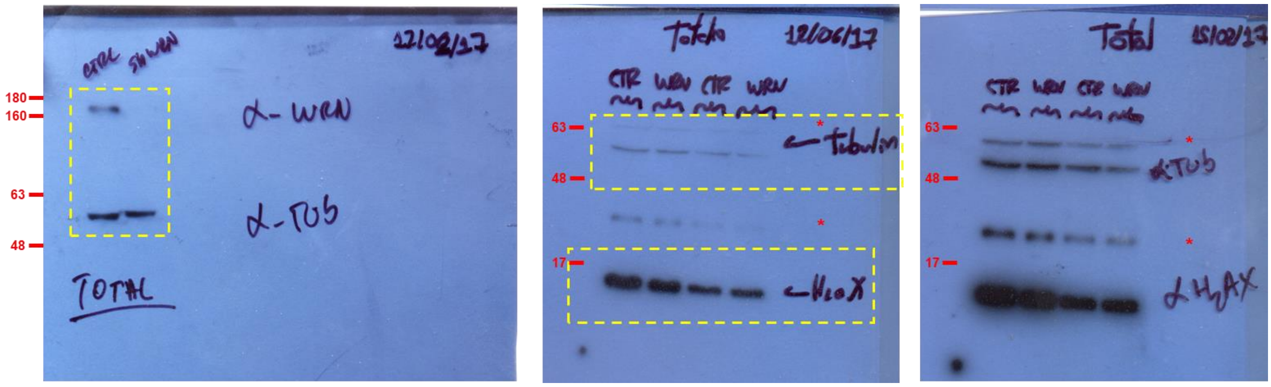

Original file 14 Original WB of **Supplementary Figures 1D and 1E**. The HRP chemiluminescent signals were captured by using X-ray film. The yellow dotted-line squares denote the cropped areas used to generate the Figures. *The asterisk denotes unspecific cross-reactivity. Molecular weight marker are shown.

<u>Western blot of cell fractionation assay by sucrose density gradient</u>

Supp. Fig 2B

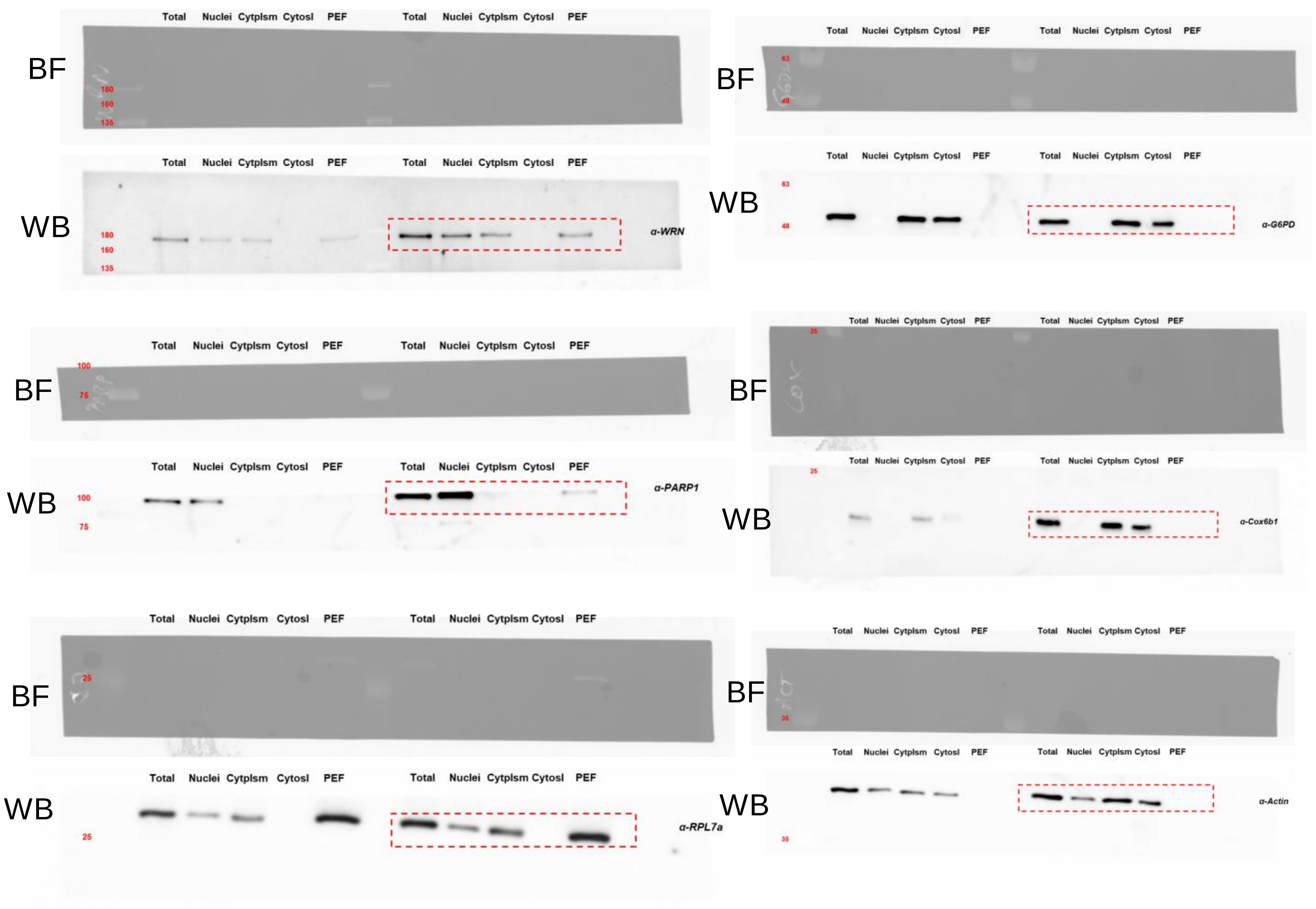

<u>Original file 15</u>. Original immunoblot of **Supplementary Figure 2B**. Molecular weight markers are shown for each membrane. The HRP chemiluminescent signals were captured by ChemiDoc with a CCD camera from Bio-Rad. The red dotted-line squares denote the cropped areas used to generate the Supplementary Figures. BF, Bright field; WB, Western blot.

Western blot of mTOR and p70S6K1 in WRN-depleted HeLa cells

Supp. Fig 3

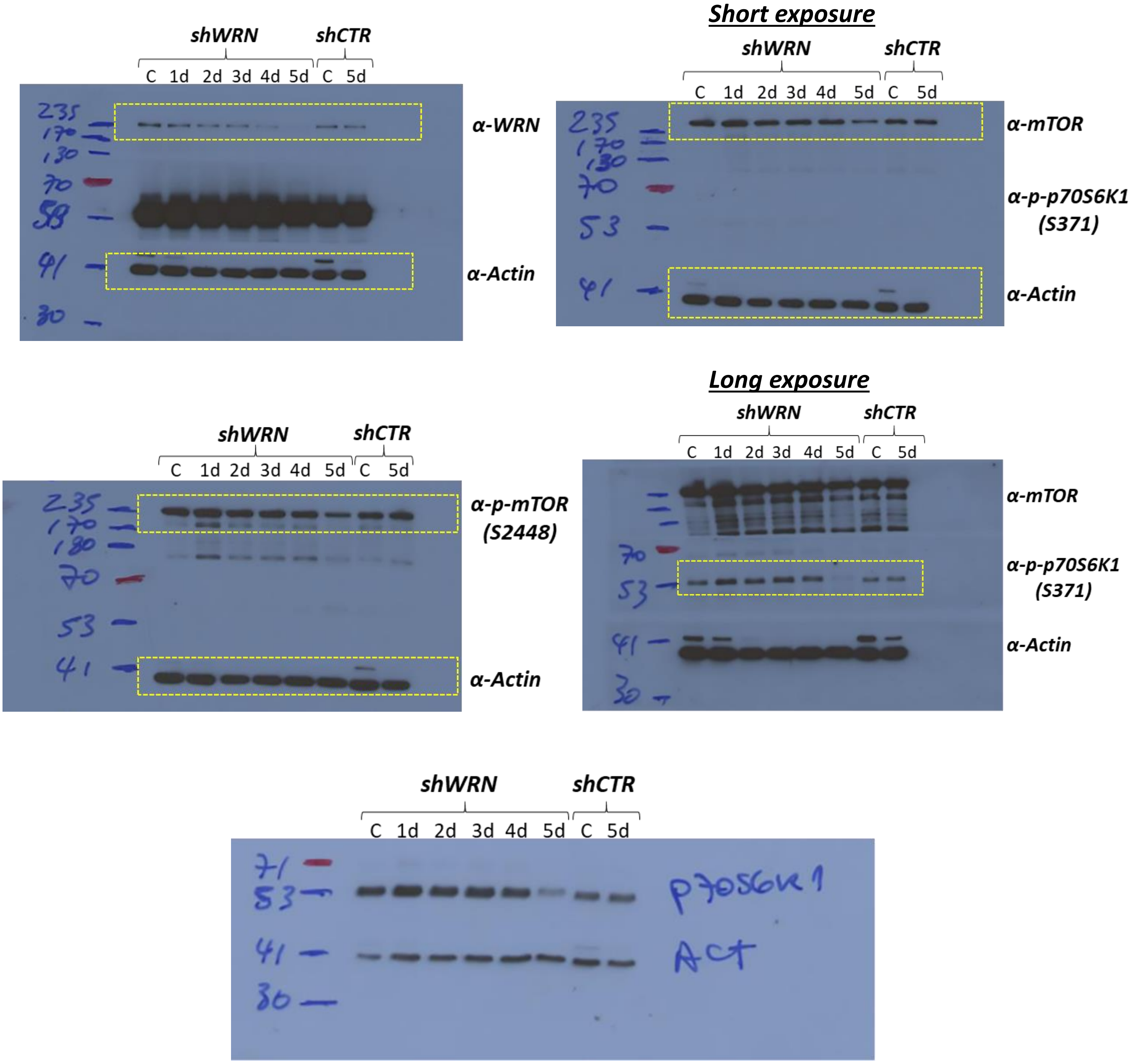

Original file 18. Original images of **Supplementary Figure 3**. The HRP chemiluminescent signals was captured by using X-ray film. The yellow dotted-line squares denote the cropped areas used to generate the Supplementary Figure. Molecular weight marker are shown.
