# Supplementary figures and images for: WRN modulates translation by influencing nuclear mRNA export in HeLa cancer cells
Source: BMC Mol Cell Biol. 2020 Oct 14;21:71. doi: 10.1186/s12860-020-00315-9 (PMC7557079; doi:10.1186/s12860-020-00315-9)

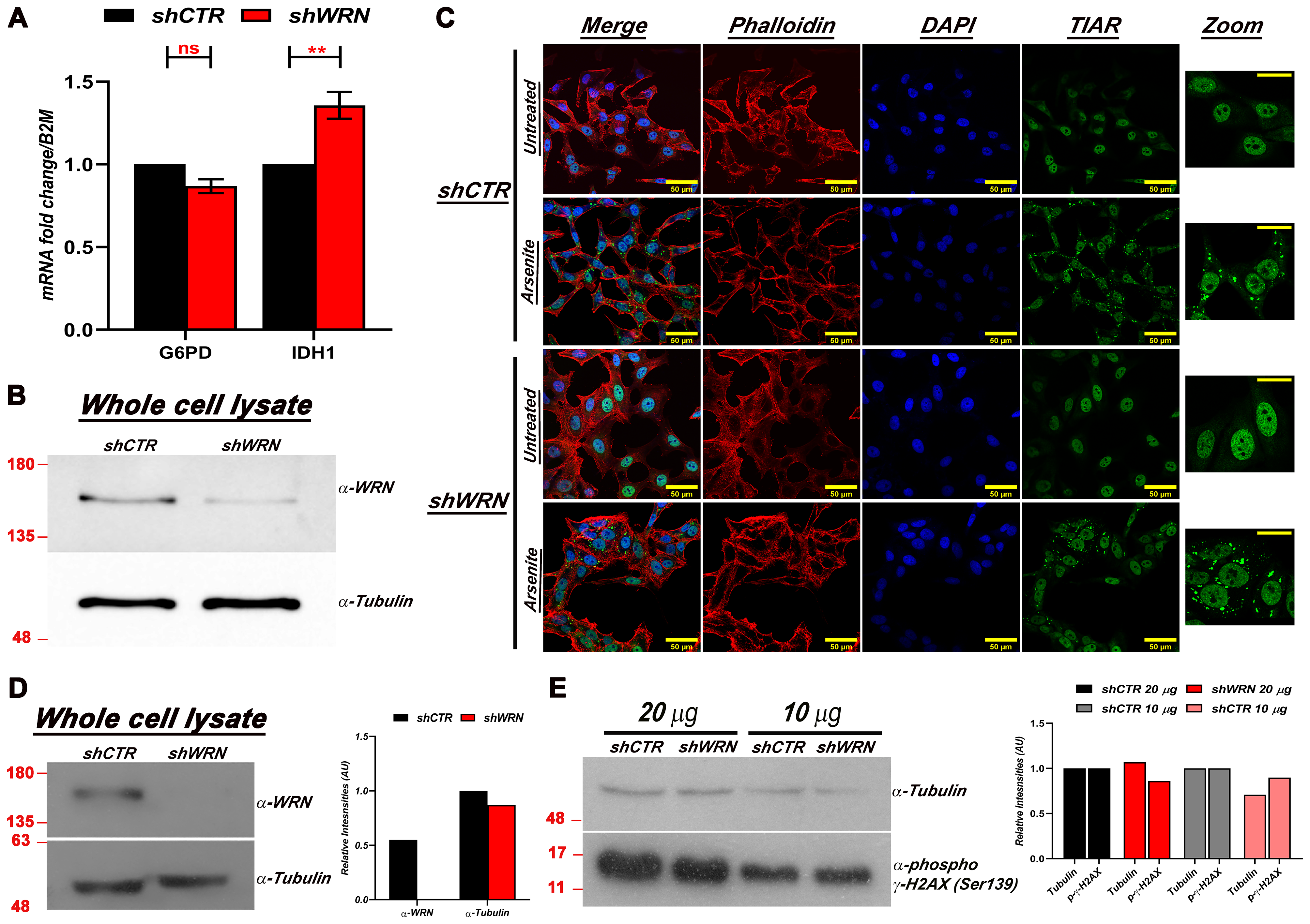

Supplement: Supplementary file 2 — Additional file 2: Supplementary Fig. 1. RT-qPCR analysis of metabolic genes, stress granules formation and oxidative DNA damage. (A) Quantitative RT-qPCR analysis of G6PD and IDH1 using B2M as the internal control were performed as described in [25]. The results of four biological replicates are plotted using GraphPad Prism. Two-way ANOVA followed by Sidak’s multiple comparisons test was used to calculate the significance. The error bars represent the mean ± SEM (n = 4). ** p value < 0.005; ns, no significant differences. (B) Western blot analysis showing WRN depletion in HeLa cells that were used for the SG formation experiment. Molecular size markers (in KiloDaltons) are shown. (C) WRN-depleted and control HeLa cells were seeded in an 8-well chamber slide. After fixation and permeabilization, the cells were incubated with the respective antibodies (see Supplemental Information and Supplementary Table 1) and counterstain solutions. The samples were analyzed by immunofluorescence using confocal microscopy. Treatment with 3 mM sodium arsenite for 2 h was used to induce stress granule formation in both cell lines (Scale bar = 50 μm). Expanded boxed regions are shown on the right (Scale bar = 20 μm). (D) Representative Western blot analysis showing reduced levels of WRN after dox treatment. (Right panel) bands quantification. (E) The extracts were assayed for protein content using the Bradford method and same amount of proteins were loaded on a polyacrylamide gel. The samples were probed for phosphor-γ-H2AX and tubulin was used as the loading control. Two different amounts of extracts were used to better visualize potential changes in phosphor-γ-H2AX. (Right panel) bands quantification. Molecular size markers (in KiloDaltons) are shown. [file 12860_2020_315_MOESM2_ESM.png]

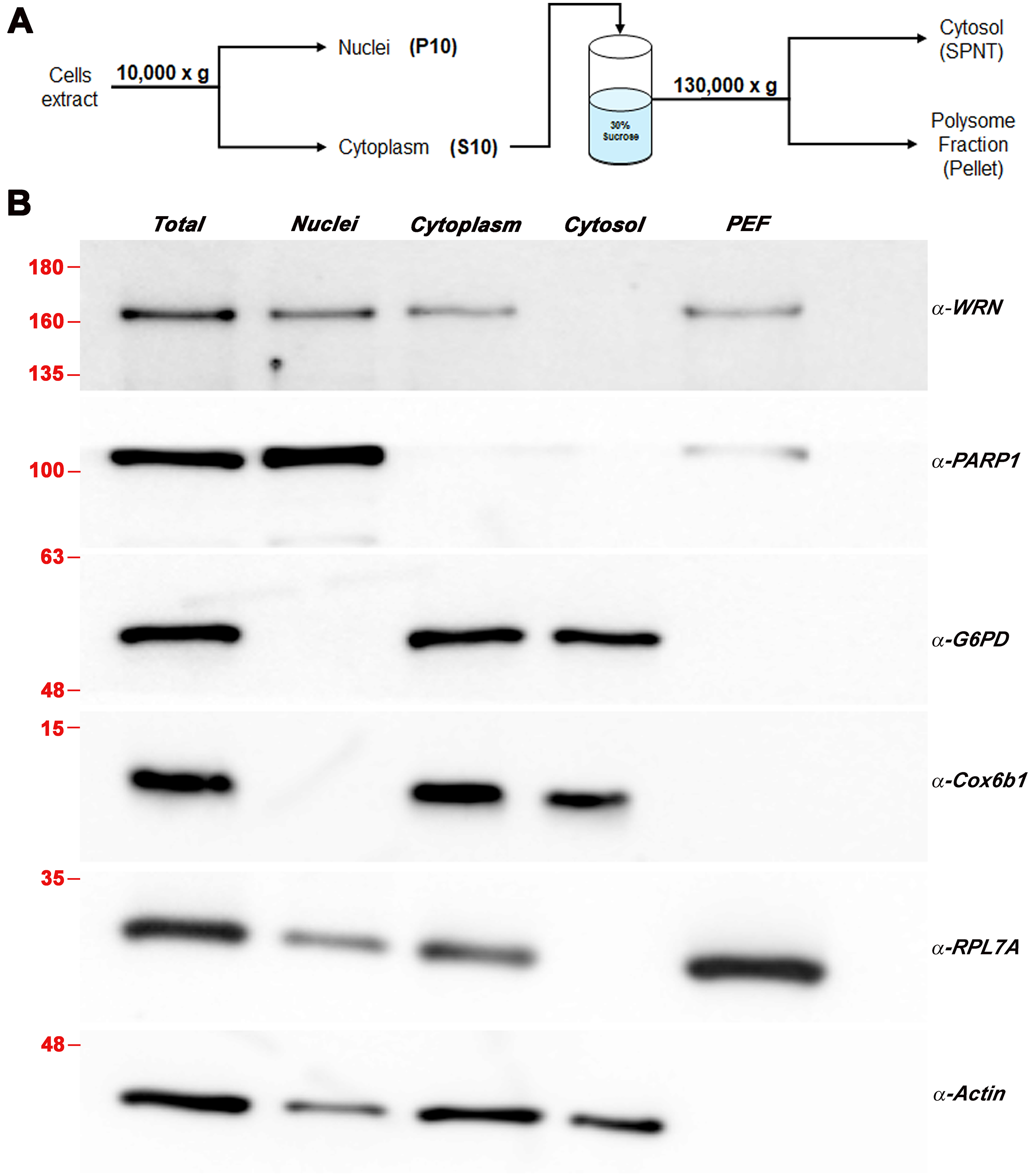

Supplement: Supplementary file 3 — Additional file 3: Supplementary Fig. 2. Nuclear/cytoplasmic fractionation and polysomes purification. Differential centrifugation followed by ultracentrifugation on a 30% sucrose cushion bed was used to generate five fractions. (A) A schematic representation of the procedure is shown. (B) Equal volumes of each fraction were loaded onto each lane. No cross contamination was observed in the fractions using PARP1 (nuclear), G6PD (Cytosol), Cox6b1 (mitochondria), RPL7a (ribosomes) and actin. The lack of detection of any of these markers in the Polysome Enriched Fraction (PEF) indicates the purity of this fraction which was used for the analysis of the 5.8S, 18S and 28S RNA by qPCR (Fig. 2c). This experiment was performed several times with identical results. [file 12860_2020_315_MOESM3_ESM.png]

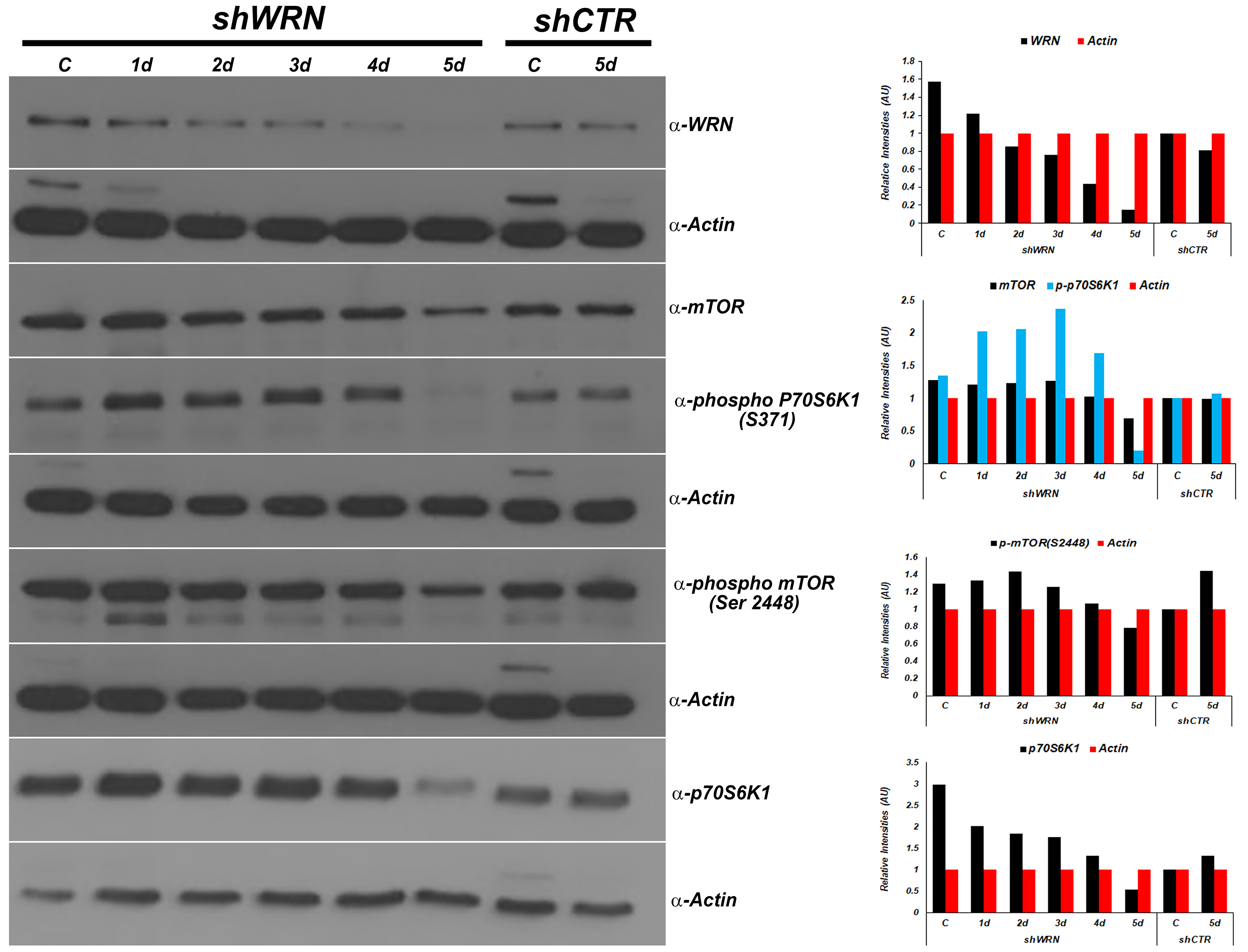

Supplement: Supplementary file 4 — Additional file 4: Supplementary Fig. 3. Analysis of mTOR and its downstream target P70S6K1 in WRN depleted and control HeLa cells. Western blot analysis of the dox time course experiment in shWRN and shCTR HeLa cells. Whole cell extracts were resolved by SDS-PAGE and immunoblotted against the indicated antibodies. The same extracts were loaded in three different gels and actin was used as a control in each blot. Bands intensities were quantitated using Image J and plotted into a graph. [file 12860_2020_315_MOESM4_ESM.png]
