# Supplementary material for: WRN modulates translation by influencing nuclear mRNA export in HeLa cancer cells
Source: BMC Mol Cell Biol. 2020 Oct 14;21:71. doi: 10.1186/s12860-020-00315-9 (PMC7557079; doi:10.1186/s12860-020-00315-9)
Supplement: Supplementary file 5 — Additional file 5: Supplementary Table 1. List of the primary and secondary antibodies used in this study. [file 12860_2020_315_MOESM5_ESM.pdf]

Supplementary Table 1

Primary antibodies	Host	Cat #	Company	Application	RRID
anti-WRN	Rabbit polyclonal	A300-239A	Bethyl Laboraotries Inc.	WB/IP	Bethyl Cat# A300-239A, RRID:AB_2216075
anti-THOC2	Rabbit polyclonal	A303-630A	Bethyl Laboraotries Inc.	WB/IP/IHC	Bethyl Cat# A303-630A, RRID:AB_11205629
anti-THOC1	Rabbit polyclonal	A302-839A	Bethyl Laboraotries Inc.	WB/IP/IHC	Bethyl Cat# A302-839A, RRID:AB_10631042
anti-GANP	Rabbit polyclonal	A303-128A	Bethyl Laboraotries Inc.	WB	Bethyl Cat# A303-128A, RRID:AB_10895633
anti-Lamin A/C (4C11)	Mouse monoclonal	4777	Cell Signaling Technology	WB/IP/IHC/IF/F	Cell Signaling Technology Cat# 4777, RRID:AB_105457
anti-RPS3 (D50G7) XP	Rabbit monoclonal	9538	Cell Signaling Technology	WB/IP/IF	Cell Signaling Technology, Cat# 9538, RRID:AB_10622028*Updated
anti-RPS6 (5G10)	Rabbit monoclonal	2217	Cell Signaling Technology	WB/IHC/IF	Cell Signaling Technology, Cat# 2217, RRID:AB_331355*Updated
anti-RPL7a (E109)	Rabbit polyclonal	2415	Cell Signaling Technology	WB/IF	Cell Signaling Technology Cat# 2415, RRID:AB_2182059
anti-G6PD (D5D2)	Rabbit monoclonal	12263	Cell Signaling Technology	WB	Cell Signaling Technology Cat# 12263, RRID:AB_2797861
anti-IDH1 (D2H1)	Rabbit monoclonal	8137	Cell Signaling Technology	WB	Cell Signaling Technology Cat# 8137, RRID:AB_10950504
anti-TIAR (D32D3) XP	Rabbit monoclonal	8509	Cell Signaling Technology	WB/IP/IF/F	Cell Signaling Technology Cat# 8509, RRID:AB_10839263
anti-THOC4/ALY (D3R4R)	Rabbit monoclonal	12655	Cell Signaling Technology	WB/IP/IF	Cell Signaling Technology Cat# 12655, RRID:AB_2797980
anti-NCBP1/CBP80 (D7Z2Z)	Rabbit monoclonal	24964	Cell Signaling Technology	WB/IP	Cell Signaling Technology Cat# 24964, RRID:AB_2798891
anti-eIF4E	Rabbit monoclonal	2067	Cell Signaling Technology	WB/IP/IHC	Cell Signaling Technology Cat# 2067, RRID:AB_2097675
anti-UAP56	Rabbit polyclonal	GTX55829	GeneTex	WB	GeneTex, Cat# GTX55829, RRID:AB_2827406
anti-TAP (G12)	Mouse monoclonal	SC-28377	Santa Cruz Biotechnology	WB/IF/IP	Santa Cruz Biotechnology Cat# sc-28377, RRID:AB_627611
anti-Cox6b1 (C-3)	Mouse monoclonal	SC-393233	Santa Cruz Biotechnology	WB/IF/IP	Santa Cruz Biotechnology Cat# sc-393233, RRID:AB_2814984
anti-Tubulin (10D8)	Mouse monoclonal	SC-53646	Santa Cruz Biotechnology	WB/IF/IP	Santa Cruz Biotechnology Cat# sc-53646, RRID:AB_630403
anti-Actin (C-11)	Goat polyclonal	SC-1615	Santa Cruz Biotechnology	WB/IF	Santa Cruz Biotechnology Cat# sc-1615, RRID:AB_630835
anti-CRM1 (C1)	Mouse monoclonal	SC-74454	Santa Cruz Biotechnology	WB/IF/IP	Santa Cruz Biotechnology Cat# sc-74454, RRID:AB_1122704
anti-PARP (46D11)	Rabbit monoclonal	9532	Cell Signaling Technology	WB/IP/IF/F	Cell Signaling Technology Cat# 9532, RRID:AB_659884
anti-TRF2 (D1Y5D)	Rabbit monoclonal	13136	Cell Signaling Technology	WB/IP	Cell Signaling Technology Cat# 13136, RRID:AB_2722641
anti-Ku70 (A-9)	Mouse monoclonal	SC-5309	Santa Cruz Biotechnology	WB/IP/IF/IHC	Santa Cruz Biotechnology Cat# sc-5309, RRID:AB_628453
anti-mTOR	Rabbit polyclonal	2972	Rabbit polyclonal	WB/IP	Cell Signaling Technology Cat# 2972, RRID:AB_330978
anti-phospho-mTOR (S2448)	Rabbit polyclonal	2971	Cell Signaling Technology	WB	Cell Signaling Technology Cat# 2971, RRID:AB_330970
anti-p70S6k	Rabbit polyclonal	9202	Cell Signaling Technology	WI/IP	Cell Signaling Technology Cat# 9202, RRID:AB_331676
anti-phospho-p70S6K1 (S371)	Rabbit polyclonal	9208	Cell Signaling Technology	WB	Cell Signaling Technology Cat# 9208, RRID:AB_330990
anti-phospho-Histone H2A.X (S139) (20E3)	Rabbit monoclonal	9718	Cell Signaling Technology	WB/IHC/IF/F	Cell Signaling Technology Cat# 9718, RRID:AB_2118009
**Secondary antibodies**	**Host**	**Cat #**	**Company**	**Application**	**RRID**
anti-rabbit IgG-HRP	Mouse monoclonal	SC-2357	Santa Cruz Biotechnology	WB	Santa Cruz Biotechnology Cat# sc-2357, RRID:AB_628497
anti-mouse IgG-HRP	Goat polyclonal	SC-2005	Santa Cruz Biotechnology	WB	Santa Cruz Biotechnology Cat# sc-2005, RRID:AB_631736
anti-goat IgG-HRP	Donkey polyclonal	SC-2020	Santa Cruz Biotechnology	WB	Santa Cruz Biotechnology Cat# sc-2020, RRID:AB_631728
anti-Rabbit IgG (H+L) Alexa Fluor 488	Donkey polyclonal	A-21206	ThermoFisher Scientific	IF	Thermo Fisher Scientific Cat# A-21206, RRID:AB_2535792
anti-Goat IgG (H+L) Alexa Fluor 594	Donkey polyclonal	A-11058	ThermoFisher Scientific	IF	Thermo Fisher Scientific Cat# A-11058, RRID:AB_2534105

Application Key: **WB**-Western blot, **IP**-Immunoprecipitation; **IHC**-Immunohistochemistry; **IF**-Immunofluorescence; **F**-Flow Cytometry.
