# Supplementary material for: WRN modulates translation by influencing nuclear mRNA export in HeLa cancer cells
Source: BMC Mol Cell Biol. 2020 Oct 14;21:71. doi: 10.1186/s12860-020-00315-9 (PMC7557079; doi:10.1186/s12860-020-00315-9)
Supplement: Supplementary file 6 — Additional file 6 Supplementary Table 2. List of the primers for qPCR used in this study. [file 12860_2020_315_MOESM6_ESM.pdf]

Supplementary Table 2

Gene	Sequences
Tubulin	Forward: 5-ATGCGTGAGTGCATCTCCATC-3
	Reverse: 5-GCCAAGTGACAAGACCATTGG-3
Actin	Forward: 5’-GCGGGAAATCGTGCGTGACATT-3’
	Reverse: 5’-GATGGAGTTGAAGGTAGTTTCGTG-3’
IDH1	Forward: 5’-CGGACTCTGTGGCCCAAGGG-3’
	Reverse: 5’-AGTCCCGTGGGCAGCCTCTG-3’
G6PD	Forward: 5’-TGCCCCCGACCGTCTAC-3’
	Reverse: 5’-ATGCGGTTCCAGCCTATCTG-3’
GAPDH	Forward: 5’-CTTTGACGCTGGGGCTGGCA-3’
	Reverse: 5’-GGCTGGTGGTCCAGGGGTCT-3’
RPS6	Forward: 5’-GCAAATCTGAGCGTTCTCAAC-3’
	Reverse: 5’-CATACTGGCGGACATCATCTT-3’
RPL7a	Forward: 5’-TCCTGCGATTAACCAGTTCAC-3’
	Reverse: 5’-TTAACTCCTGCTCGAAGGACA-3’
RPS3	Forward: 5’-GGCATCTTCAAAGCTGAACTG-3’
	Reverse: 5’-CTGGAAAGCCAAACCTCTTCT-3’
rRNA 28S	Forward: 5’-GGGTTTTAAGCAGGAGGTGTC-3’
	Reverse: 5’-AACCTGTCTCACGACGGTCTA-3’
rRNA 18S	Forward: 5’-CGGCGACGACCCATTCGAAC-3’
	Reverse: 5’-GACGGGGAATCAGGGTTCGATTC-3’
rRNA 5.8S	Forward: 5’-GACTCTTAGCGGTGGATCACTC-3’
	Reverse: 5’-ATCGACACTTCGAACGCACT-3’
