# Supplementary material for: WRN modulates translation by influencing nuclear mRNA export in HeLa cancer cells
Source: BMC Mol Cell Biol. 2020 Oct 14;21:71. doi: 10.1186/s12860-020-00315-9 (PMC7557079; doi:10.1186/s12860-020-00315-9)
Supplement: Supplementary file 7 — Additional file 7. Supplemental Information. [file 12860_2020_315_MOESM7_ESM.docx]

***Supplemental Information***

***WRN modulates translation by influencing nuclear mRNA export in HeLa cancer cells***

Juan Manuel Iglesias-Pedraz*; Diego Matia Fossatti Jara; Valeria del Carmen Valle-Riestra Felice; Sergio Rafael Cruz Visalaya; Jose Antonio Ayala Felix and Lucio Comai.

* To whom correspondence should be addressed: Juan Manuel Iglesias-Pedraz, PhD., ORCID iD: 0000-0003-0070-8116. Laboratorio de Genética Molecular y Bioquímica, Universidad Científica del Sur, Lima 15842, Perú; Telephone: +51 (1) 610 6400 Anx 572; e-mail: [jmiglesi71@gmail.com](mailto:jmiglesi71@gmail.com)

***Supplemental Experimental Procedures***

***Inducible Tet-ON/Tet-OFF lentiviral shRNA vectors***

To downregulate WRN protein expression, we generated a conditional shRNA vector construct targeting the 3´-UTR of WRN mRNA. The shWRN was designed using the sequence ranging from 5272-5297 (22nt) of accession number AF091214.1 (NM_000553.5), obtained from GenBank, NCBI (GenBank, RRID:SCR_002760). Them, complementary oligonucleotides were annealed and cloned into the pENTT-miRc2 vector. The sequences are: WRN-3´UTRf: 5′-GCCTACGTGAGTACATCACCTA-3´. miR-shRNA vectors were then generated by *in vitro* recombination between pENTT-miRc2 and pSLIK-Neo (RRID: Addgene_25735) or pSLIK-Hyg (Addgene #25737) using the Gateway LR Clonase Enzyme Mix Kit (Invitrogen, Carlsbad, CA, USA). For the production of recombinant lentiviruses, we followed the protocol previously described by Shin, K. J. *et al* 2006(1). We performed lentiviral infections as previously described in Li, B. et al. 2014(2). The infected cells were then washed twice with PBS 1X, and after 24 hours subjected to selection using 400 μg/mL geneticin (G418) and 200 μg/mL hygromycin for seven days.

***Metabolic labeling using ^35^S-met/cys***

These experiments were performed as described by Bonifacino, J. S. 2001(3). First, three pairs of tissue culture dishes with shCTR and shWRN HeLa cells were treated with doxycycline for three days. Two days after induction, cells were subcultured into two new dishes to avoid overcrowding and incubated for an additional day in the presence of doxycycline. On the third day of incubation, one pair of the dishes was used to confirm WRN depletion by Western blot while the remaining two pairs of dishes were used for metabolic labeling. For the metabolic labeling experiments, HeLa cells were washed twice before being incubated in pre-warmed pulse-labeling medium (methionine/cysteine-free DMEM) at 37 °C for 30 min to deplete the intracellular pool of methionine/cysteine. The medium was then removed and 3 mL of freshly prepared labeling solution containing 0.1 mC/mL TRANS35S-LABEL MP Biomedicals, USA, made in pulse-labeling medium) was added to the cells. Cells were then incubated for 15 min before being rinsed with ice-cold PBS for three times. Cells were then collected by trypsinization, counted by Trypan-Blue (TB) exclusion method, and equal number of cells were lysed in Lysis buffer (25 mM TRIS-HCl pH 8.0; 100 mM NaCl; 0.1% TX-100; 0.1% SDS; 1 mM EDTA; 1 mM DTT and PIC (1/100 v/v). The cell lysates were centrifuged at 20,000 x g at 4 °C, and the extracts were used to measure *de-novo* protein synthesis by SDS-PAGE or immunoprecipitation assays.

***Immunoprecipitation of ^35^S-met/cys labeled proteins***

^35^S-met/cys labeled extracts were prepared as described above. For the immunoprecipitation assay, we used the same concentration of antibodies against G6PD, Ku70, and Tubulin in each reaction. Reactions were incubated overnight at 4°C in a roller and immune complexes were captured using Protein A/G PLUS-Agarose (Santa Cruz Biotechnology, sc-2003,) following the manufacturer’s instructions. The immunoprecipitated complexes were resolved on 10% PAA and stained with CBB followed by vacuum drying. Finally, the dried gels were scanned for IgG visualization and then exposed to phosphoscreen and developed using a PharosFX molecular imager (BIO-RAD).

***Nuclear-Cytoplasmic fractionation and preparation of the Polysome Enriched Fraction (PEF)***

All the reagents and buffers were RNase/DNase free or prepared in the DEPC-treated water, autoclaved and kept on ice or in the cold room for at least 1 hour before the experiment. For PEF we follow the protocol described by Masek, T. et al 2011(4). Before harvesting, cells were treated with 100 μg/mL of cycloheximide (CHX) for 10 min at 37 °C. The dishes with the cells were then transferred to an ice bed and washed twice with ice-cold PBS containing 100 µg/mL CHX. Cells were scraped off the dishes with a cell lifter and collected in PBS buffer containing 100 µg/mL CHX. After centrifugation at 1,850 x g, the cell pellets were rinsed in hypotonic buffer (10 mM HEPES-KOH pH 7.6; 1.5 mM MgCl_2_; 10 mM KCl; 1 mM PMSF; 5 mM NaF; 5 mM β-GP; 1 mM DTT; 200U RNase Inhibitor Murine (40 U/μL); EDTA-Free Protein inhibitor cocktail PIC (1/100 v/v) and 100 μg/mL CHX) and spun down immediately for 5 min at 4 °C at 1,850 x g. The supernatant was transferred to a new 1.5 mL ice-cold tube, and the cell pellets were resuspended again in hypotonic buffer and incubated for 15 min on ice. NP-40 (0.1 % final concentration) was then added and incubated with occasional tube inversion for an additional 10 min or until more than 95% of cells where positive for TB staining. The homogenates were centrifuged at 1,850 x g for 15 min at 4 °C to separate the cytoplasmic fraction from the nuclei. The cytoplasmic fraction was centrifugated again at 10,000 x g for 15 min at 4°C to ensure complete elimination of the nuclei. The pelleted nuclei were resuspended in hypotonic buffer supplemented with 420 mM NaCl and incubated on ice for additional 45 min to extract nuclear proteins. For the preparation of the Polysome Enriched Fraction, the cytoplasmic fraction, obtained as described above, was overlaid on a 1 mL sucrose cushion (30%) prepared in the same hypotonic buffer(4, 5). The sample was centrifuged using Optima LE-80K (Beckman Coulter) in an SW55-Ti pre-chilled rotor at 130,000 x g for 2.5 hours. The supernatant was discarded and the pellet was quickly rinsed with hypotonic buffer and then resuspended in 2X protein loading buffer for protein analysis or resuspended in the same buffer (100 µL) for RNA extraction using TRIzol (Thermo Fisher) following the manufacturer’s instructions.

***Gene expression analysis by RT-qPCR***

All the reagents and buffers were RNase/DNase free or prepared in the DEPC-treated water that was autoclaved and kept on ice or in the cold room for at least 1 hour before being used for the experiments. Total and ribosomal RNAs were prepared using TRIzol (Thermo Fisher) following the manufacturer instructions. After three days of dox treatment, the cells were either treated or not with cycloheximide (CHX, 100 µg/mL) for 10 min in the CO_2_ incubator. For the isolation of total RNA, untreated cells were scraped off the dish and washed twice with ice-cold PBS. The cell pellet was then resuspended in a small volume of 1X PBS, and 0.8-1 mL of TRIzol was directly added to the sample. Ribosomal was isolated from the Polysome Enriched Faction prepared as described above. The PEF was resuspended in TRIzol for RNA isolation. RNA quality was assessed by visualization on formaldehyde-agarose gels and RNA concentration was calculated using a NanoDrop spectrophotometer (ND-1000, NanoDrop Technologies, Wilmington, Delaware). We routinely treated two micrograms of each RNA sample with DNase (Turbo DNase AM-1907-ThermoFisher Scientific) following the manufacturer’s instructions. The RNA samples were subjected to PCR reactions using Taq MasterMix (abm; G013) to ensure the lack of genomic DNA contamination. RNAs were retrotranscribed to generate cDNAs using the kit OneScript cDNA Synthesis SuperMix from abm (G452), following the manufacturer’s instructions. For qPCR reactions, 10 to 100pg of each freshly synthesized cDNA was analyzed using BrightGreen 2X qPCR MasterMix-R (with ROX) from abm, according to the manufacturer’s instructions and StepOne Real-Time PCR (Applied Biosystems) thermocycler. For each qPCR reaction, a specific set of primers against the target was generated using Primer3: WWW primer tool online(6). Each set of primers was validated using the Blastn suite from NIH(7). All the primers were designed with an estimated melting temperature of 60°C. GAPDH or 18S were used as internal controls.

***Immunofluorescence and stress granule (SG) formation assay***

For the immunofluorescence assay, cells were seeded in a 100 mm plates (Celltreat) and grown in media containing dox (1.5 µg/mL). After two days of dox treatment, cells were trypsinized and transferred to 8-well culture cell slides (Lab-Tek® II Chamber Slide) at a concentration of 3x10^4^ cells/well. An aliquot of the cells was left in 100 mm plates with media containing dox to monitor WRN downregulation by Western blot. The 8-well cell culture slides were cultured for an additional day in media containing dox. Before cell fixation, cells in a set of wells were treated with Sodium Arsenite (3 mM) for 2 hours. Cells were then rinsed with 1X PBS (RT), fixed for 10 min at RT in a freshly prepared PFA solution (4% in 1X PBS) and then permeabilized with 0.5 % TX-100 (in 1X PBS). The blocking step was carried out with blocking buffer (2.5% BSA and 1% Normal Serum raised in the same host as the secondary antibody in 1X PBS) for 1 hour at room temperature. The rabbit anti-TIAR primary antibody diluted in the blocking buffer was added to the samples and incubated overnight at 4 °C. The next day, cells were washed with 1X PBS three times for 5 min with agitation and incubated in the dark for 1 hour at RT with a combination of Donkey anti Rabbit IgG Alexa Fluor 488-conjugated secondary antibody and Alexa Fluor-594 Phalloidin (Supplemental Table 1). The samples were washed five times with 1X PBS for 5 min each and then mounted using VECTASHIELD HardSet Antifade Mounting Medium with DAPI (H-1500) from Vector Labs. Images were acquired with a confocal laser scanning microscope (Nikon C2).

***Co-Immunoprecipitation assays***

We prepared ten 100mm dishes containing HeLa cells at 75% confluency. On the day of the experiment, the dishes were incubated in fresh media for additional 2 hours. Cells were washed with ice-cold 1X PBS DEPC-treated twice and scraped off. The cells were centrifuged at 1,500 x g for 5 min at 4 °C and pellets were lysed in Lysis Buffer (50 mM HEPES-KOH pH 7.5; 150 mM NaCl; 50 mM KAc; 0.5% TX-100; 0.5% NP-40; 2 mM EDTA; 10% Glycerol; 1 mM PMSF; 1 mM NaF; 1 mM Na_3_VO_4_; 1 mM β-GP and PIC (1/100 (v/v) prepared in DEPC-treated water) for 10 min on ice. An aliquot of this extract is saved as input. The lysates were divided into two equal volumes aliquots and transferred to a new tube. One aliquot was treated with 10 mM of Ribonucleoside Vanadyl Complex (RVC) to protect RNAs (NEB S1402S) and the other with 250U of Benzonase (250U; SC-391121). Both tubes were incubated on ice for 40 min and centrifuge at 14,000 x g for 30 min at 4 °C. Supernatants were transferred to new ice-chilled tubes and separated in equal volumes aliquots according to the number of antibodies to be used including the control isotype. To each tube, we added Lysis buffer containing either RVC or Benzonase to bring the volume to one milliliter. An aliquot of each sample was used as Input and to assess the RNA integrity by stained formaldehyde-agarose gel. Antibodies were added to the reactions and incubated overnight at 4 °C in a roller mixer. The next day, three hundred twenty microliters of Protein G Magnetic Beads were equilibrated by washing three times in Lysis buffer and blocked in the same Lysis Buffer containing 2% of acetylated BSA for 1 hour at 4 °C. Thirty microliters of blocked beads were added to each reaction and incubated for 2 hours at 4 °C in a roller mixer. Before washing the beads, we removed an aliquot to assay RNA integrity as described above. The beads were then washed three times with Lysis buffer and three times with Wash buffer (Lysis buffer with 300 mM NaCl) using a magnetic rack. The immunocomplexes were eluted with 10 µL of 0.2 mM Glycine pH 2.8 twice, 10 min each, at RT and the elutes neutralized by adding 3 µL of 1M Tris-HCl pH 8. The same volume of 2X protein loading buffer was then added to each reaction, mixed and heated at 95 °C for 5 min. The samples were then analyzed by Western blot.

***mRNA Fluorescence In Situ Hybridization (FISH)***

RNA FISH was performed as previously described by Viphakone, N. et al, 2012(8) with some modifications. The cells with shWRN or shCTR were grown on 100 mm dishes in media containing dox for two days. The cells were then transferred to 8-well chamber slides or coverslips for an additional day in presence of dox. The cells were washed two times with 1X PBS-DEPC at room temperature to remove unattached and debris cells and fixed for 10 min at room temperature with 4% paraformaldehyde that was freshly prepared in 1X PBS-DEPC. Cells were washed three times in 1X DEPC-treated PBS and permeabilized for 10 min at RT with 0.5% TX-100 prepared in 1X DEPC-treated PBS. After two washes with DEPC-treated PBS and once with 2X SSC-formamide pH 7.0 (30 mM sodium saline citrate, 300 mM sodium chloride and 20% formamide), the cells were pre-hybridized for 2 hours at 37 °C with hybridization solution (2X SSC, 20% formamide, 1 μg/μL tARN, 1% acetylated BSA (B2518, Sigma-Aldrich), 2mM RVC, 10% dextran sulphate and 1 µg/µL salmon sperm). The samples were then incubated overnight at 37 °C with hybridization solution containing 1 ng/μl Cy3 labelled oligo (dT)_50_ (Gene Link) in the hybridization oven. Next day, the cells were washed three times with 2X SSC-formamide at 37°C and once with 0.5X SSC at RT. Finally, the samples were rinsed three times at RT with 1X DEPC-treated PBS (DAPI was included in the last wash as counterstain) before mounting on glass slides or cover by coverslip for microscopy analysis. We include RNase, Benzonase and DNase treatments as controls for the specificity of the signal. More than 300 cells for each cell sample in three biological replicates were counted for the statistical analysis. The resulting images were used to measure the pixel intensity of the Oligo(dT)-Cy3 signal along a 5 pixels in width line drawn across the length of the cell(9).

***mRNA pull down assays***

We prepared fifteen 100mm dishes with HeLa cells at 75% confluency. On the day of the experiment, we replaced the media and incubated the cells for an additional 2 hours. The cells were then washed with ice-cold 1X PBS-DEPC twice and scraped off with a cell lifter. The cells were centrifuged at 1,500 x g for 5 min at 4 °C and resuspended in 1 mL of 1X DEPC-treated PBS. The cell suspensions were equally divided into two RNase/DNase-free pre-chilled 1.5 mL tubes and pelleted again. Cells were lysed for one hour on ice with Lysis Buffer (25 mM Tris pH7.5; 5 mM MgCl_2_; 0.5% NP40; 150 mM NaCl; 1 mM DTT; 2 mM PMSF; PIC (1/100 (v/v)); 5 mM β-GP and 1 mM NaF) prepared in DEPC-treated water. 10 mM of RVC or 100 µg/mL RNase A (G117, abm) were added to the aliquots. After 30 min of incubation, the crude extracts were clarified at 15,000 x g for 30 min at 4 °C and supernatants were transferred to a new RNase/DNase-free ice-chilled tube. A small aliquot was used to monitor RNA integrity on formaldehyde-agarose gels. During the clarification step, we prepared 100 µL of Oligo d(T)_25_ Magnetic Beads (NEB S1419S) by washing and blocking in Lysis Buffer containing either RVC or RNase A/DNase-Free, and 5% acetylated BSA for 1 hour at room temperature in a roller mixer. The beads (50 µL for each tube) were combined with clarified extract and incubated for 2 hours at 4 °C in a roller mixer. Before starting the washes, an aliquot was used to assay RNA integrity on formaldehyde-agarose gels. The magnetic beads were washed three times for 10 min each with Lysis Buffer (containing RVC or RNase A) using a magnetic stand. Then the beads were eluted with loading buffer and heated at 95 °C for 5 min. The samples were magnetically separated, and the supernatant recovered into a new tube. These samples were resolved by SDS-PAGE and probed against the indicated antibodies.

***References***

1. Shin KJ, Wall EA, Zavzavadjian JR, Santat LA, Liu J, Hwang JI, et al. A single lentiviral vector platform for microRNA-based conditional RNA interference and coordinated transgene expression. Proc Natl Acad Sci U S A. 2006;103(37):13759-64.

2. Li B, Iglesias-Pedraz JM, Chen LY, Yin F, Cadenas E, Reddy S, et al. Downregulation of the Werner syndrome protein induces a metabolic shift that compromises redox homeostasis and limits proliferation of cancer cells. Aging Cell. 2014;13(2):367-78.

3. Bonifacino JS. Metabolic labeling with amino acids. Curr Protoc Cell Biol. 2001;Chapter 7:Unit 7 1.

4. Masek T, Valasek L, Pospisek M. Polysome analysis and RNA purification from sucrose gradients. Methods Mol Biol. 2011;703:293-309.

5. Belin S, Hacot S, Daudignon L, Therizols G, Pourpe S, Mertani HC, et al. Purification of ribosomes from human cell lines. Curr Protoc Cell Biol. 2010;Chapter 3:Unit 3 40.

6. Rozen S, Skaletsky H. Primer3 on the WWW for general users and for biologist programmers. Methods Mol Biol. 2000;132:365-86.

7. Altschul SF, Madden TL, Schaffer AA, Zhang J, Zhang Z, Miller W, et al. Gapped BLAST and PSI-BLAST: a new generation of protein database search programs. Nucleic Acids Res. 1997;25(17):3389-402.

8. Viphakone N, Hautbergue GM, Walsh M, Chang CT, Holland A, Folco EG, et al. TREX exposes the RNA-binding domain of Nxf1 to enable mRNA export. Nat Commun. 2012;3:1006.

9. Viphakone N, Cumberbatch MG, Livingstone MJ, Heath PR, Dickman MJ, Catto JW, et al. Luzp4 defines a new mRNA export pathway in cancer cells. Nucleic Acids Res. 2015;43(4):2353-66.
